# Voltage vs. Ligand I: Structural basis of the intrinsic flexibility of S3 segment and its significance in ion channel activation

**DOI:** 10.1080/19336950.2019.1674242

**Published:** 2019-10-24

**Authors:** Daniel Balleza, Mario E. Rosas, Sergio Romero-Romero

**Affiliations:** a Departamento de Química ICET, Universidad Autónoma de Guadalajara, Zapopan Jalisco, Mexico; b Facultad de Medicina, Departamento de Bioquímica, Universidad Nacional Autónoma de México, 04510 Mexico City, Mexico. Current address: Department of Biochemistry, University of Bayreuth, Bayreuth, Germany

**Keywords:** Local flexibility, voltage sensor, S3 segment, channel activation

## Abstract

We systematically predict the internal flexibility of the S3 segment, one of the most mobile elements in the voltage-sensor domain. By analyzing the primary amino acid sequences of V-sensor containing proteins, including Hv1, TPC channels and the voltage-sensing phosphatases, we established correlations between the local flexibility and modes of activation for different members of the VGIC superfamily. Taking advantage of the structural information available, we also assessed structural aspects to understand the role played by the flexibility of S3 during the gating of the pore. We found that S3 flexibility is mainly determined by two specific regions: (1) a short NxxD motif in the N-half portion of the helix (S3a), and (2) a short sequence at the beginning of the so-called *paddle* motif where the segment has a kink that, in some cases, divide S3 into two distinct helices (S3a and S3b). A good correlation between the flexibility of S3 and the reported sensitivity to temperature and mechanical stretch was found. Thus, if the channel exhibits high sensitivity to heat or membrane stretch, local S3 flexibility is low. On the other hand, high flexibility of S3 is preferentially associated to channels showing poor heat and mechanical sensitivities. In contrast, we did not find any apparent correlation between S3 flexibility and voltage or ligand dependence. Overall, our results provide valuable insights into the dynamics of channel-gating and its modulation.

## Introduction

The most prominent characteristic of ion channels is their ability of gating. Probably, the simplest model to understand this property is to consider the transition between two distinct functional states: the “closed state” (commonly at resting) and the “open state”. At the nanoscale, the transition between these two states involves a structural rearrangement that is elicited by a plethora of physical and chemical stimuli. In “voltage-gated” channels, the electrical potential difference across the cell membrane is sensed by a V-sensor that facilitates the pore opening through conformational changes characterized by an outgoing and twist movement of the S4 segment [1–4]. In general terms, this behavior is conserved in distant proteins such as the voltage-sensing phosphatase of *Ciona intestinalis*, Ci-VSP, or the Hv1 proton channel, where S4 also moves upward upon depolarization [5,6]. This kind of activation has been considered ancestral in ion channel evolution and mainly was present in prokaryotic K^+^-selective pores more than 3000 myr ago [7]. On the other hand, quite diverse “ligands”, including protons, divalent ions, and complex molecules such as membrane signaling lipids, purine cyclic nucleotides, and other metabolites can interact with several ion channels inducing the opening of the pore. Activation by ligand binding has also been dated very early in the prokaryotic lineage [8] and it can be achieved through specific interactions between the ligand and binding sites, which are mostly located in cytoplasmic modules as well as in the voltage sensor domain itself [9–11]. However, there is no exclusive stimulus to activate a particular type of ion channel; contrarily, there is increasing evidence showing that for many ion channels, more than one kind of “activator” can trigger their opening. For example, from a thermodynamic point of view, temperature should be considered a potential factor controlling gating, because higher temperature could in principle favor the movement of specific segments that are involved in the conformational change occurring during the pore opening, or contribute to reducing the restriction exerted by other parts of the same protein during such process [12].

Conformational flexibility is an inherent property of proteins and, in the case of the V-sensor, one of the premises is that local flexibility is important for the conformational changes involved in channel gating. According theory, as protein flexibility controls the thermodynamic rate of conformational changes, there is a subtle equilibrium between flexibility and rigidity of the structure to compensate thermal fluctuations [13,14]. It has been determined that, for example, in L-type Ca^2+^ channels (Ca_V_1) the flexibility of the S3-S4 linker of domain IV determines the substantial conformational changes that VSD undergoes upon activation and it play a crucial role in determining the gating properties of this channel [15,16]. Both theoretical and experimental evidence also indicate that the C-terminal half of S3 (S3b), which is directly attached to the segment S4 through the S3-S4 linker (S3-S4L), has more structural mobility than the almost static S1 to S3a segments [4,17–20]. NMR spectroscopic data also indicate that, once activated, this helix-turn-helix (S3b-L-S4) *paddle*-motif turns to adopt a more rigid substructure, showing high stability in the ps-ns time scale [21].

In K**_V_** channels, this motif has been described as a structural element conferring important internal flexibility to the VSD, in such a way that it is thought to be capable of assuming different conformations, at least in the archaeal KvAP channel [1]. Although this model of gating has been extrapolated to more distant channels, such as the canonical *Shaker* (from *Drosophila*) and their mammalian homologues (K**_V_**1.2), evidence also indicate that motion of the VSD could involve other parts of the protein and be inconsistent with the *paddle*-motion mechanism [18,22–24]. Thus, although the nature of S4 motion is still not clear probably due to the model used, to date the most accredited mechanism proposes that, in V-dependent channels, upon depolarization, S4 segments move outward carrying charged residues across the membrane field, facilitating the opening of the pore through an electromechanical coupling which moves the lower gate [23,25]. Local mobility of specific segments into the VSD has also been described in HCN channels, where the opening is instead coupled to a downward motion of S4 in response to hyperpolarization [26].

In TRP channels, which are weakly voltage-sensitive but strongly activated by stimuli as diverse as heat, cold, and several ligands, gating is also achieved through conformational changes facilitated by local flexibility. These motions are associated with specific regions of the VSD, including the S3 segment, as well as linkers S3-S4L and S4-S5L [9,27–29]. In CNG channels, which are virtually voltage-independent, the opening of the pore has evolved in strict dependence on the cyclic nucleotide-binding through conformational changes involving the S4-S5L [30].

These channels belong to the superfamily of voltage-gated ion channels (VGIC) and exhibit, overall, the same modular design. Their structures are characterized by a V-sensor domain (VSD) composed of four transmembrane (TM) α-helices, the segments S1 to S4, and a pore domain (PD) encompassed by the segments S5 to S6, which suggest common ancestry [7,31,32]. The modular nature of this superfamily is also revealed by the fact that both the VSD and the PD form functional proteins separately; i.e. each domain works as a self-cooperative unit. This is the case of Hv1 proton channels as well as the voltage-sensing phosphatase (VSP), where the VSD, instead of opening a pore, it activates an enzyme [6,33]. Regarding the PD, the basic building block of inwardly rectifying K^+^-channels (K_ir_) and two-pore domain K^+^-channels (K2P) exhibit the simplest transmembrane topology, resembling the S5-to-S6 segment present in 6TM voltage-gated ion channels. Thus, these channels are equivalent to the PD with an intervening reentrant P-loop including the selectivity filter (SF), but lacking the V-sensor. As in the case of TPC channels [34], K2P channels differ from K_ir_ channels because they assemble as a “dimer of dimers” with each subunit containing four TM segments (TM1–TM4) required to form a “tetrameric” K^+^-channel [35].

The aim of this work was then to systematically predict the internal flexibility of the second more mobile element of the VSD after S4, the S3 segment, from primary amino acid sequence in every family of V-sensor containing proteins, including the Hv1 and the VSP families. With this information, we have proposed to establish some relationship between this intrinsic property and the voltage- or ligand-dependent activation in those proteins. Likewise, with the advantage of having available structural information of most representatives of each subfamily integrating this superfamily, we contrast our predictive analysis with structural aspects that allow us to better understand the role played by local flexibility in the basic aspect of the activity in these proteins: their ability of gating. Notably, we found a good correlation between S3 flexibility indices and the sensitivity to temperature so that if the channel exhibits high sensitivity to heat, local S3 structures become more rigid. On the other hand, highly flexible S3 profiles were found in channels fully dependent on ligand or frequently associated to auxiliary subunits. In those cases, the temperature coefficient (Q_10_) tends to be minimum. Regarding voltage dependence, this property is amply distributed in practically all members of this superfamily with subtle differences between specific groups of ion channels. Additionally, we evaluated the flexibility profiles of S3-S4 hairpin in order to explore the important contribution of the S3-S4L to the VSD activation as well as the whole flexibility of the complete VSD. Our results indicate that there are some sites of important local flexibility inside the V-sensor; however, the previously mentioned correlation regarding S3 flexibility and its relationship with the dependence on ligand and temperature is, in general, maintained. Likewise, we further explore an interesting relationship between S3 rigidity and stretch-activation reported for some members of this heterogeneous group of membrane proteins. In the companion paper, we hypothesize on the evolution of the VSD and describe the functional implications of the high flexibility present in CNG channels as well as we also report a structural model for the CNG-like channel AqK from the thermophilic bacterium *Aquifex aeolicus*, one of the oldest species of bacteria.

## Material and methods

### Voltage-gated ion channel (VGIC) sequence database

Our analysis was based on the classification of Yu and Catterall [31] for the so-called VGL-chanome of V-gated and V-gated–like ion channels. Therefore, 11,170 non-redundant sequence entries from different groups of eukaryotes and selected prokaryotes were used to represent the complete superfamily. The following databases were searched to build our VGIC DataBase: GenBank, RefSeq, UniProtKB/SwissProt, and the PDB. Different well-studied protein sequences, mainly from mammals, were used as queries in BLAST-P searches using their non-redundant protein sequences database. Thus, we organized all the sequences in 23 different groups and the analyzed sequences were organized as follows: 1630 members representing the K_V_ family (K_V_1 to K_V_4 or *Shaker-Shab-Shaw-Shal* subfamilies); 782 sequences representing the EAG family (K_V_10 to K_V_12, KCNH or ether-à-go-go); 484 sequences for the K_V_7 family (KCNQ1-5); 868 members of the Ca^2+^- and Na^+^-dependent potassium channel families (K_Ca_ and K_Na_); 1671 members of the transient receptor potential (TRP) channels including subfamilies TRPV (Vanilloid), TRPM (Melastatin) and TRPA (Ankyrin) proteins; 652 members of the cyclic nucleotide-gated (CNG) channels including subunits CNGA1 to CNGA4, CNGB1 and CNGB3; 359 members of the hyperpolarization-activated, cyclic nucleotide-gated channels comprising four subfamilies (HCN1-4).

The dataset includes eukaryotic two- and four-domain 6TM voltage-gated cation channels: 1166 members representing the voltage-gated sodium channels (Na_V_1 family) and 982 members of the voltage-gated calcium channels including the L-type subfamily (Ca_V_1.1 to Ca_V_1.4), the Ca_V_2 subfamily (Ca_V_2.1 to Ca_V_2.3) which mediate P/Q-type, N-type, and R-type Ca^2+^ currents, respectively, and the Ca_V_3 subfamily (Ca_V_3.1 to Ca_V_3.3) which mediate the T-type Ca^2+^ currents. Regarding the two-pore channel (TPC) family, since they may represent an intermediate stage in the evolutionary transition between 6TM potassium channels and four-domain voltage-gated Ca^2+^ and Na^+^ channels [32,34], we decided to include a broad spectrum of metazoan organisms taking as query sequences the ones of the starlet sea anemone (*Nematostella vectensis*), sea urchin (*Stronglylocentrotus purpuratus*), sea squirt (*C. intestinalis*) as well as *Homo* and *Arabidopsis* from which the crystal structure is known [36]. This family is composed by 1061 sequences.

VGIC dataset also includes 495 sequences of the voltage-gated proton channel (Hv1) family and 244 members of the voltage-sensitive phosphoinositide phosphatase VSP/TPTE family. In these two families, orthologues from diverse basal metazoan organisms were included but in the case of the Hv1 family, in addition to representatives from *C. intestinalis* and diverse mammals, we also included homologous sequences to several unicellular organisms such as *Emiliania huxleyi, Coccolithus pelagicus* ssp. braarudii as well as zebrafish (*Danio rerio*). Finally, we included 776 orthologs of the large-conductance mechanosensitive channel (MscL) from Gram-negative and Gram-positive bacteria. This family was considered in our study since the S1-TM1 linker of MscL is highly conserved and contains the same NxxD motif found in segment S3 of voltage sensors and because it has been proposed as reminiscent of a common ancestral sensor for both voltage and membrane tension [37].

### Multiple sequences alignment and phylogenetic analysis

Alignments were assembled using the Clustal-W algorithm [38] with the Gonnet protein weight matrix. Location of TM segments studied was compared using the aligned profiles with the Uniprot server and contrasted them with previous reports on literature. Sequence alignments of interest were graphically represented using the public WebLogo server (http://weblogo.threeplusone.com) and, to estimate intrinsic local flexibility, we generated three different consensus sequences to include the contribution of the amino acid composition as a standard deviation. The overall height of each sequence logo indicates the sequence conservation at that position, while the height of symbols (amino acids) represent the relative frequency of each residue at that position. Color identifiers are as follow: hydrophobic residues (red); aromatic rigid side-chains (dark red); α-Helix stabilizing alanine (salmon); small and flexible side-chains (orange); polar uncharged and flexible side-chains (gray); highly flexible positive (dark blue) and negative charged (teal); α-Helix breaker proline (purple); Cys and His (black).

### Estimation of flexibility indices and electrostatic interactions

Atomic temperature factors (B-factors) obtained during structural determination are a trustworthy measure of the flexibility of each residue in the protein. We used normalized B-values to estimate the average flexibility indices for the desired segment of each generated consensus sequence. Since the flexibility of a residue depends on the nature of its side-chain as well as on their neighbors at the primary sequence level, we estimated the intrinsic flexibility of a residue in accordance to calculations considering these factors, and that have been reported elsewhere [39,40]. In such a way, we report the average of the B-factors (the mean B-factor, mBf) which reflect the flexibility profile of the studied segment. In some cases, we included the relative solvent accessibility (RSA) index as a measure of the average contributions of each amino acid to the folding stability. Salt bridges were assessed according to the distance between donor Lys (N^ζ^ atom) or Arg (N^ζ^, N^η1^ and N^η2^) in S4 and the two acceptor carboxyl oxygens in the Asp residue of the NxxD motif at S3. A cutoff distance of ≤ 4.0 Å was considered as a salt bridge according to Xu et al. [41].

### Online supplemental information

Our supplemental material provides figures depicting local S3 flexibility profiles and their S3-S4 structural information for selected members of the VGIC not included in the main text. Consensus S3 sequences and relevant information of the VGIC Dataset is also available. This information is accessible online.

## Results

Since the so-called *paddle* is proposed to move at the protein-lipid interface but has been formally described only in the KvAP channel, we wonder if there is another flexible sequence motif common to all of these members of the VGIC superfamily. Technically, the *paddle* motif starts at the helix breaker Pro99 in the KvAP channel (Pro322 in *Shaker*) [1]. However, about three turns before in the S3 helix the NxxD motif is, in fact, more conserved among very diverse families of sensor domains and includes an important negatively charged residue (D316 in *Shaker*) that is part of the so-called CTC or charge transfer center, also named the gating pore through which the movement of S4 is facilitated during the activation-deactivation cycle [20,42]. This residue is highly conserved among almost all members of the VGIC superfamily, including the Hv1 proton channels and the VSPs (*see* ref. 37 and cited literature therein). Interestingly, this motif has also been found near the cytoplasmic membrane before the TM1 segment in the mechanosensitive channel of large conductance MscL in the orthologues from *Mycobacterium tuberculosis* and *Staphylococcus aureus* (Suppl. Figure 1). The NxxD motif could represent a sensor in mechanosensation since mutant channels shift the activation curves to membrane tensions less than those required to activate the wild-type MscL [43]. As this motif is also a functional component of the V-sensor in voltage-dependent channels, mutations of this region are likewise critical in channel gating [27,44,45]. Interestingly, in MscL orthologues, two helical turns below the NxxD motif, a conserved Gly residue (G24 in *M. tuberculosis*) forms a girdle surrounding the narrowest portion of the channel lumen [46]. This residue is positioned at the equivalent location of the *paddle*-motif in V-dependent channels (Suppl. Figure 1).

**Figure 1.**
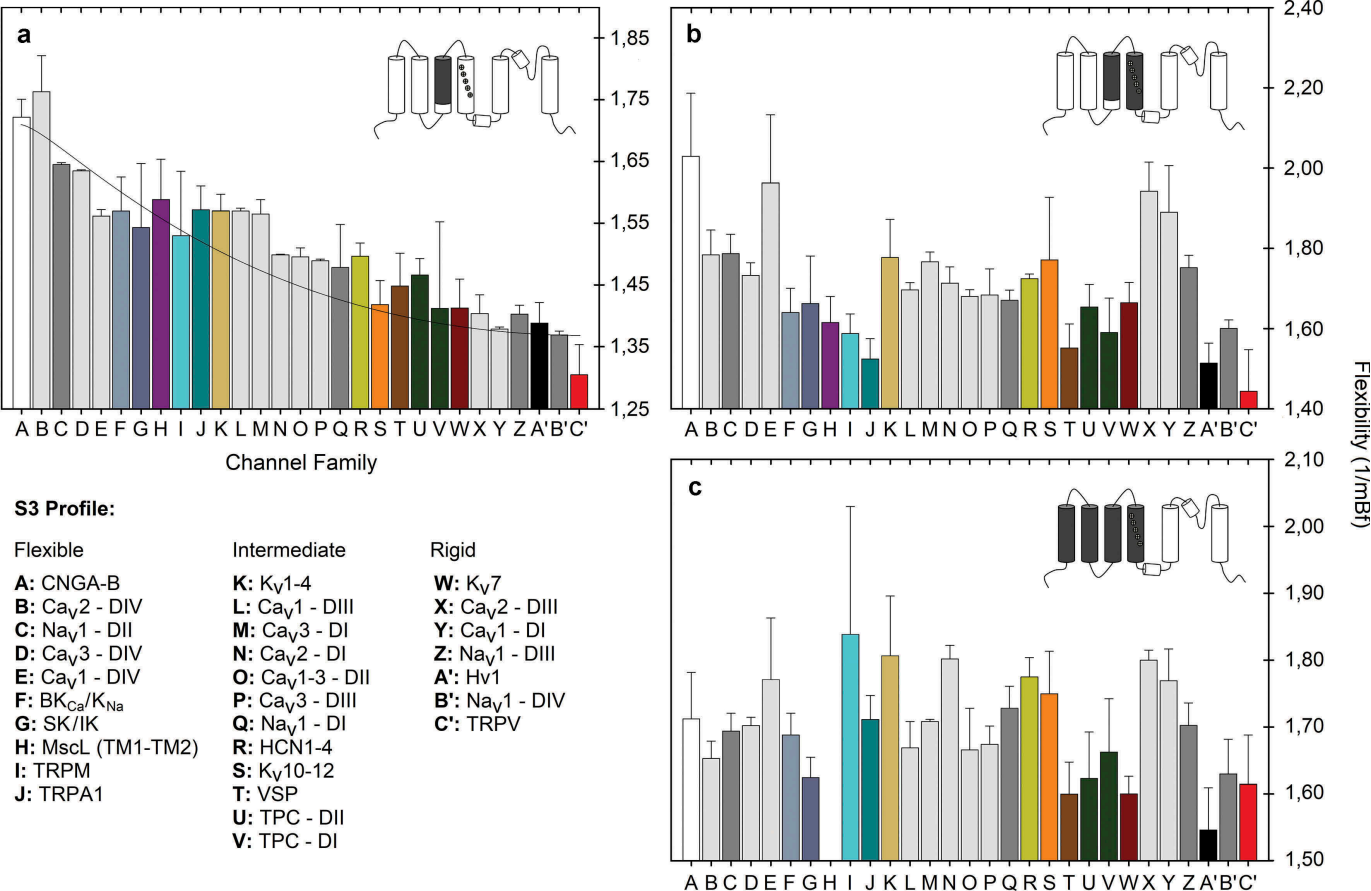
Distributions of mean B-factor values of specific segments in members of the VGIC superfamily. Panel **A** shows results for segment S3 starting at the NxxD motif. Panel **B** shows results including the S4 segment and panel **C** quantify the mean B-factor of the whole voltage-sensor in each family. The computations are based on consensus sequences and standard deviation representing the second and third more conserved residue by position. The VGIC dataset includes 11,170 non-redundant sequence entries. The curve is a modified single-exponential fit for the data using the following equation (*R*^2^ = 0.95): f = a • exp[b/(x + c)].

### Local flexibility

Based on the high sequence similarity exhibited by the V-sensors studied here, we identified the NxxD motif at the S3 segment in each ion channel family of the VGIC dataset. Each alignment was then analyzed separately in order to create 29 consensus sequences, corresponding to this segment – one for each family – starting at the NxxD motif and finishing at the C-end portion of the α-helix as reported on the Uniprot server, or according to published reports (Table S1). Then, using two different prediction algorithms for side-chain flexibility (*see* Material and Methods), we calculated the local flexibility of three different segments inside the VSD: (1) from the NxxD motif at S3 to the C-terminal end of that segment; (2) from NxxD of S3 to the C-end of S4, and (3) from the N-end of S1 to the C-end of S4, encompassing the whole VSD. Figure 1 shows plots of the flexibility indices representing the distribution of the normalized B-factors for each segment. Distribution was not homogeneous and does not follow a normal distribution so we categorized flexibility indices according to three functional groups of ion channels, belonging to three main flexibility profiles of segment S3: (1) channels exhibiting a flexible profile represented by CNG, BK_Ca_, SK/IK, TRPM, TRPA1, the domain IV of Ca_V_1-3 channels and the domain II of Na_V_ channels; (2) channels with an intermediate flexibility profile including members of K_V_1-4, HCN and K_V_10-12 families, the voltage-sensitive phosphatase family, TPC channels, the domain I of Ca_V_2-3 and Na_V_1 channels, the domain II of Ca_V_1-3 and domain III of Ca_V_1 and Ca_V_3 families; and (3) channels with a rigid profile for S3 which interestingly includes K_V_7 (KCNQ), Hv1 proton channels, the heat-activated capsaicin receptor TRPV family as well as the domain I of Ca_V_1, the domain III of Ca_V_2 and domains III-IV of Na_V_ channels.

When we compare the flexibility profiles of segment S3-S4 and the whole VSD, previous S3 pattern disappear for some members but important information emerges or remains constant. For example, we noted that the TRPM family to which the cold/menthol-activated TRPM8 belongs [47], exhibits great diversity in flexibility profiles at the level of the whole VSD. On the other hand, CNG and domain IV of Ca_V_ channels are highly flexible while K_V_7, Hv1, TRPV, domain III of Ca_V_2, domain I of Ca_V_1 and domain III and IV of Na_V_ channels are always rigid independently of the analyzed segment. Interestingly, in sodium channels, voltage-sensing domains III and IV, firmly established as participating in fast inactivation [48,49] show a rigid profile if they are compared with domains DI and DII, which are mainly associated to channel activation [50]. Finally, by comparing subfamilies K_V_1 (*Shaker*), K_V_2 (*Shab*), K_V_3 (*Shaw*) and K_V_4 (*Shal*) with members of the KCNH channel family (K_V_10, K_V_11 and K_V_12) we found that the *paddle* motif as well as the length of S3-S4 linker, which is longer in K_V_1-4 channels, are the main responsible conferring diverse flexibility profiles to the segment S3. This makes K_V_1-4 family more flexible than K_V_10-12 channels, with an amino acid composition and overall architecture significantly distinct [51] (Suppl. Figure 2 and Suppl. Figure 3). Nevertheless, the *paddle*-motif is not so well conserved into the VGIC superfamily as the NxxD motif (*see* Suppl. Table 1).

**Figure 2.**
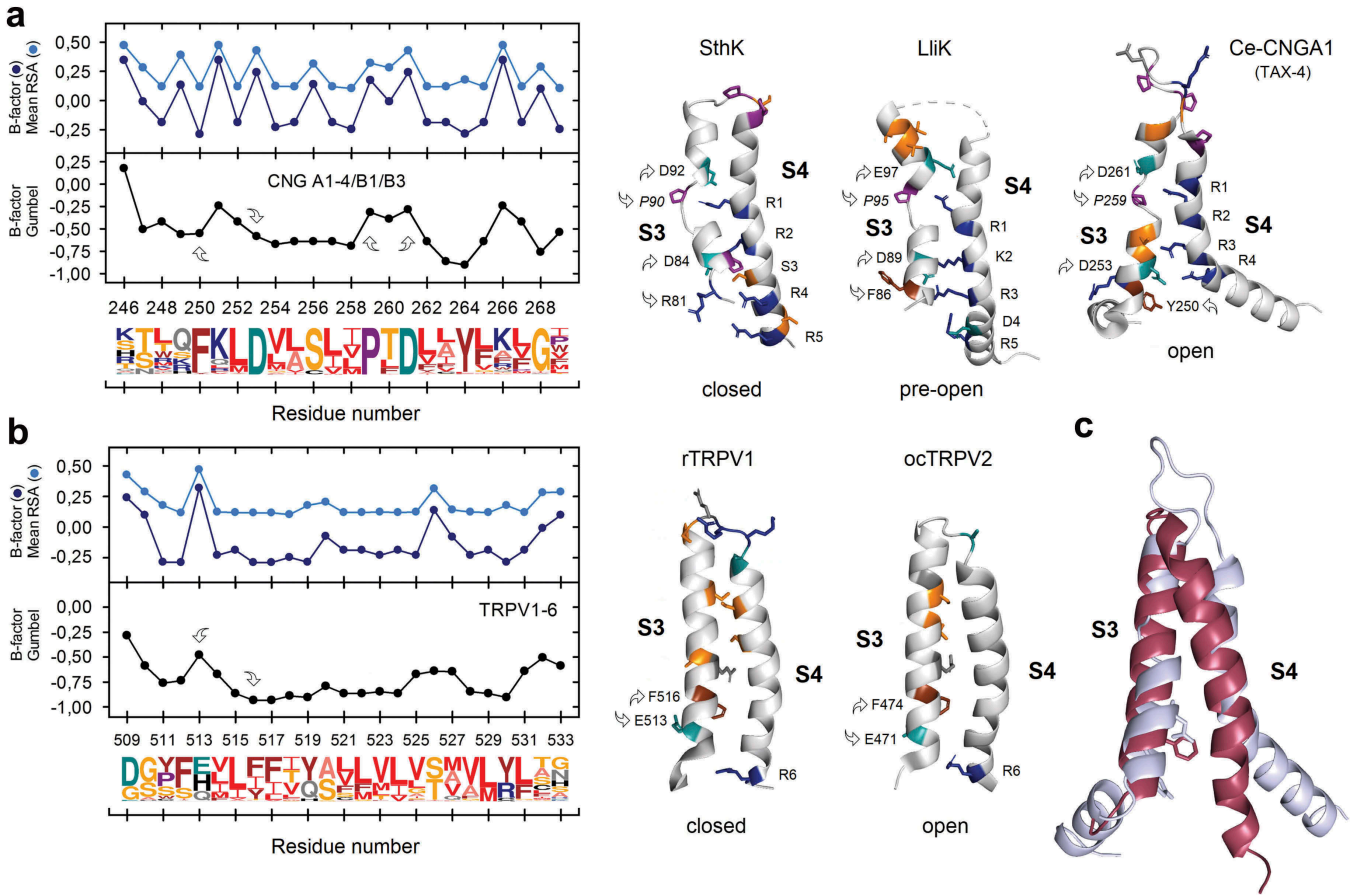
(**A**) Predicted S3 flexibility profile for the CNG family (CNGA1-4, CNGB1 and CNGB3). Two sequence motifs with high intrinsic flexibility and strongly conserved (arrows) are implicated in the conformational arrangement of S3 segment in members of this family which includes the prokaryotic CNG channels from *Leptospira licerasiae* (LliK; PDB ID: 5V4S) and *Spirochaeta thermophila* (SthK; PDB ID: 6CJQ) as well as the eukaryotic CNG channel from *Caenorhabditis elegans* (TAX-4; PDB ID: 5H3O). (**B**) Flexibility profile for the TRPV channel family which was the lower one for the VGIC Dataset. Note that the *paddle*-motif is not present here and, in consequence, segment S3 is comparatively straighter and more rigid than the one present in CNG channels. The structure of TRPV1 from rat (PDB ID: 5IRZ) and TRPV2 channel in complex with resiniferatoxin from rabbit, *Oryctolagus cuniculus* (PDB ID: 6BWJ) are included for comparison. (**C**) Superimposed high-resolution structures of TAX-4 and TRPV2 proteins. Sequence logos were obtained using the Weblogo 3.0 server. Numbering corresponds to TAX-4 and TRPV1 protein sequences respectively. Arrows denote the location of the NxxD and the *paddle*-motifs. Residues forming part of these motifs and those with flexible side chain are shown in color. As proline is an “α-helix breaker”, it is depicted in italics. See Material and Methods for colors code.

**Figure 3.**
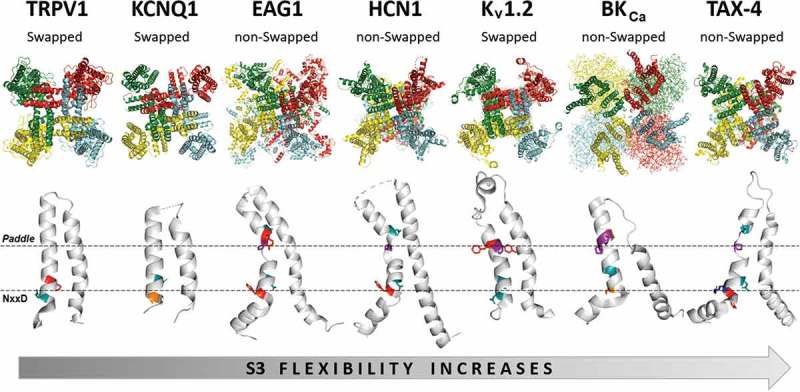
Comparison of domain-swapped and non-domain-swapped architectures of members of the VGIC superfamily as a function of their S3 flexibility. Each complete subunit is shown in a different color. Only the main contributors to local flexibility are shown with colors highlighting the location of NxxD and *paddle* motifs in the S3 segment. Structures viewed from the membrane plane (*top*) are shown for TRPV1 (PDB ID: 5IRZ), KCNQ1 (PDB ID: 5VMS), EAG1 (PDB ID: 5K7L), HCN1 (PDB ID: 5U6P), K_V_1.2 (PDB ID: 3LUT), BK_Ca_ (PDB ID: 5TJI) and TAX-4 (PDB ID: 5H3O).

As members of the CNG family exhibit one of the more flexible S3 segments in the VGIC dataset, this prompted us to contrast such profiles structurally with the more rigid counterpart, which was found in members of the TRPV family. Figure 2 compares both the flexibility profiles and local S3-S4 topologies in two of the recent available structures corresponding to the cyclic-nucleotide-gated channel formed by TAX-4, a CNG-A isoform from *Caenorhabditis elegans* in the cGMP-bound open state (PDB: 5H3O) and the crystal structure of the TRPV2 channel from rabbit in complex with resiniferatoxin (PDB: 6BWJ). Superposition of these structures using the analogous NxxD motif (FxxD) as a reference shows a deviating RMSD of 2.8 Å and a prominent kink of S3 segment from TAX-4 one helical turn below connecting it to S2. This feature is absent in TRPV channels. Consistent with a highly-flexible profile for S3 in CNG channels, we confirmed that the *paddle* motif (P-T-D), which start segment S3b, is structurally disordered in both prokaryotic (LliK, PDB: 5V4S and SthK, PDB: 6CJQ) and eukaryotic (TAX-4) CNG channels (Figure 2A). Moreover, structure of segment S4 significantly departs from the canonical one present in K_V_ and TRPV channels since it does not adopt a continuous helical structure, but it breaks up into three sub-segments, which have been named as S4a (loop), S4b (a 3_10_ helix) and S4c (an α-helix) [52]. It is also important to mention that, according to these authors, although segment S4 in CNG channels possess regularly spaced Arg residues throughout, this structural arrangement senses only a fraction of the transmembrane voltage field, making CNG channels practically insensitive to the membrane potential. On the other hand, in prokaryotic CNG-like channels, S4 is straighter, making those channels more responsive to changes in the membrane potential [8].

A further evident difference between CNG-TAX4 and TRPV2 channels is the length of the S3-S4 linker, which is considerably short in the rigid protein and longer in the flexible one (Figure 2C). Remarkably, the rigid nature of the V-sensor that we found here for the TRPV family is consistent with the almost null conformational change for the S1-S4 region of TRPV1 during the closed-to-open transition [28,53]. However, some other evidence also indicates that substantial conformational changes are associated with other parts of the protein, namely the S4-S5L and the pore helix in TRPV2 [54]. This localized mobility point to a fast transition through the concerted effect of the enthalpic and entropic components of gating [55], and is also consistent with the finding that the TRPV pore is dynamic and capable to adopt different conformations, exhibiting distinct conductances, as a function of the kind of agonist and exposition time [56,57]. Therefore, a link between local rigidity and significant protein motion emerges. In this regard, we infer that the α-to-π helical transitions localized in the S6 segment of TRPV3 during channel sensitization [58,59] are favored by the rigid profile present in these proteins. On the other hand, channels with a flexible profile, such as TRPM8 – where no α-to-π helical transitions during gating have been described so far – can adopt more freer conformations, and less-localized. Rather, this could be related with α-to-3**_10_** helical transitions, which result in a register change of the helix and the ensuing adaptability of the binding site to diverse ligands in order to activate the channel [60]. In CNG channels, the only cryo-EM structure for TAX-4 corresponds to the channel in the cGMP-bound fully open state, however, if one contrast the structures for SthK (closed) and LliK (pre-open) with TAX-4, it becomes evident the important role that flexibility has in these channels, particularly the *paddle* motif and the S3-S4L during the closed-to-open transition. This is not observed in the case of TRPV1 and TRPV2 (Figure 2). Notably, as in the case of TRPM8, single-molecule force spectroscopy has revealed a highly dynamic VSD in CNG channels, where S4 motion is mechanically coupled to S3 or S5 in the closed-to-open transition. This also indicates that the degree of α-helix folding in the VSD varies and change to 3**_10_** conformations [61].

The analysis carried out between the CNG and TRPV families was then applied to every one of the families that compose the VGIC superfamily. Figure 3 displays a comparison between seven solved structures of ion channels in increasing order of flexibility. These structural data show several important points: (1) the total flexibility of the segment S3 is determined mainly by the presence of the NxxD and the *paddle* motifs; (2) flexibility of the S3-S4 hairpin is strongly influenced by the length of the S3-S4L; (3) channels with a non-swapped architecture between the VSD and the PD are mainly associated with flexible S3 profiles; (4) in non-swapped channels segment S3 is kinked near to the NxxD motif; and (5) channels with low flexibility profile lack the *paddle* motif and/or the NxxD motif possesses residues with rigid side chains (Phe, Tyr, His or Cys) instead Asn or Asp, according to the classification of Zhang et al. [40]. With this information, we decided to analyze the different mechanisms of activation reported in the literature for each channel families studied here in an attempt to establish a connection linking the specific local flexibility of these proteins with their main mode of activation.

The movement of positively charged S4 segment through the electric field drives the gating of voltage-sensitive ion channels [2,3]. Therefore, we hypothesized that since S4 positive charges are the main gating carriers, specific counter-charge interactions with S1-S3 residues should be favored in K_V_, Na_V_ and Ca_V_ channels but restricted or absent in poorly voltage-sensitive channels such as members of the CNG or TRPV families. To test this and relate it with the S3 flexibility pattern, specific salt bridges between amino acid pairs involving the NxxD motif were estimated. To accomplish this aim, the available structures of K_V_1.2 (PDB code: 3LUT), EAG1 (5K7L), CNGA-TAX4 (5H3O) and TRPV1 (5IRZ) were evaluated at atomic detail. These channels were selected according to their decreasing dependences on voltage, measured as the number of elementary charges (*e***_0_**) previously reported in the literature (Table 1). For example, in K_V_1.2, Asp259, Lys306 and Arg309 are close enough to form salt bridges (D259 to K306: 2.5–3.0 Å; D259 to R309: 2.1 Å) (Suppl. Figure 2). As expected, the helix-breaker proline that defines the S3a-S3b kink in K_V_1.2 and in all members of the K_V_1-4 family confers important static flexibility to this region. In contrast, K_V_7 (KCNQ) channels lack the typical Pro residue at the *paddle* motif and they show an alternative NxxD motif having preferentially cysteine or serine instead asparagine (C/S-x-x-D). This confers a substantial rigidity to the equivalent segment (Suppl. Figure 2). A similar local rigid profile was also found in the ether-à-go-go family of K_V_ channels (KCNH) encompassing three distinct subfamilies: Eag (K_V_10), Erg (K_V_11), and Elk (K_V_12) even if they exhibit the flexible *paddle* motif with the kinked S3a-S3b design (Suppl. Figure 3).

**Table 1.** Comparison between local flexibility, voltage dependence and temperature coefficient for selected ion channels.

Channel	S3 Flexibility index(1/mBf)	S3-S4 Flexibility index (1/mBf)	S1-S4 Flexibility index (1/mBf)	Charge per channel (*e*_0_)	Q_10_ (τ_act_)	Q_10_ (τ_inact_)	References
*Shaker* K_v_	1.48	1.96	1.87	12 – 16	~ 4	7.2	[44,107,121–125]
K_v_1.2	1.61	1.86	1.82	10	*n.d.*	*n.d.*	[125]
Na^+^-channel (frog muscle)	–	–	–	~ 12	2.4	3–5.3	[126,127]
Na^+^-channel (muscle)	–	–	–	~ 12	2.3	3.4–9.1	[126,127]
Na_v_1.5 DI	1.56	1.69	1.67	~ 12	1.25	4 ; 5.8^a^	[82]
Na_v_1.5 DIV	1.51	1.62	1.68	~ 12	1.25	4 ; 5.8^a^	[82]
Ca^2+^-channel (muscle)	–	–	–	8.6 – 15^b^	~ 3.0	*n.d.*	[128,129]
Ca_v_ 1.2 DII	1.58	1.70	1.67	~ 9	*n.d.^c^*	3^d^	[129,130]
Ca_v_ 1.2 DIV	1.62	1.90	1.70	~ 9	*n.d.^c^*	3^d^	[129,130]
Ca_v_ 1.4 DII	1.66	1.68	1.65	~ 9	*n.d.^c^*	~ 19^e^	[129,130]
Ca_v_ 1.4 DIV	1.62	1.97	1.75	~ 9	*n.d.^c^*	~ 19^e^	[129,130]
K_v_7.4 (KCNQ4)	1.53	1.62	1.58	~ 8	8.3	*n.d.*	[81]
K_v_11.3	1.56	1.84	1.81	8	3.8	2.6	[131]
K_v_11.1 (hERG)	1.51	1.80	1.74	6 – 8	4.7	2.9	[131,132]
HCN2	1.63	1.73	1.80	5 – 8^f^	*n.d.*	*n.d.*	[133]
HCN1	1.62	1.73	1.78	3.75	2.9	3.1^g^	[134]
hHv1	1.40	1.49	1.50	6	6 – 9	–	[115,135]
mHv1	1.40	1.46	1.49	4	6 – 9	–	[115,136]
BK (Slo1)	1.55	1.67	1.64	2.4–4.4	*n.d.*	*n.d.*	[137–139]
ciVSP	1.52	1.63	1.66	~ 1.25	1.4	1.7 ^g^	[140]
mTRPM8	1.51	1.59	1.60	0.9	1.2	5.2–9.4 ^h^	[47,141,142]
mTRPA1	1.50	1.45	1.69	*n.d.*	2.8	–	[143]
DmTRPA1	1.47	1.54	1.72	*n.d.*	11	–	[143]
TRPV1	1.44	1.52	1.66	0.5–0.7	~ 15 – 40	1.35 ^g^	[47,55,144]
TRPV2	1.49	1.52	1.55	*n.d.*	>100	–	[64]
CNGA1	1.77	1.93	1.82	~ 0.22	n.d.	–	[145]
CNGA2	1.64	1.78	1.68	*n.d.*	2 – 6 ^i^	–	[108]

Notes for Table 1

**^a^**Q_10_ at −70mV or −50mV

**^b^**Charge per channel depends on the β subunit used. α**_1E_** and α**_1E_** β**_1a_** gave high *e***_0_** values (~15)

**^c^**However, increasing the temperature from 23°C to 37°C increases whole-cell conductance and shifts the voltage-dependence of activation to more hyperpolarized voltages [130]

**^d^**Cav1.2 inactivation kinetics are not strongly temperature-dependent [130]

**^e^**Inactivation is more sensitive to lower temperature changes, compared with those at higher temperatures (*i.e*. 23–33°C *vs*. 27–37°C [130])

**^f^**Binding of cAMP to the channels stabilizes the open pore, reducing the total gating charge from ~8 to ~5

**^g^**Q_10_ for the deactivation rate

**^h^**Q_10_ estimations for the deactivation rate, including slow and fast components

**^i^**Mean Q_10_ exhibits a maximum of ~6 only at [cGMP]/EC**_50_** near 0.1 [108]

**Figure 4.**
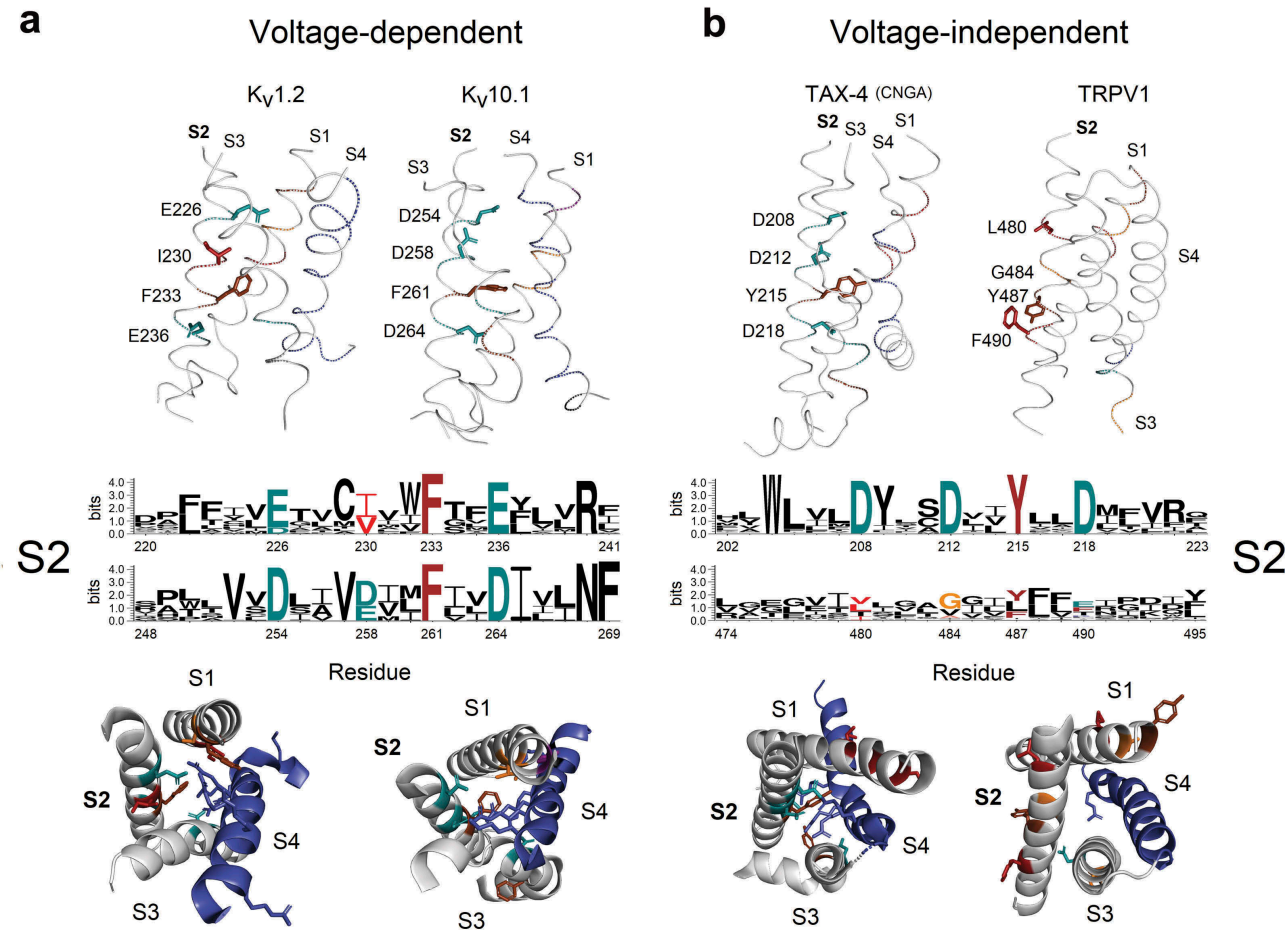
Distribution and conservation of S2 counter charges in the voltage sensor module of voltage-dependent (**A**) and independent (**B**) channels. Upper and lower panels show the refined structures of the K_V_1.2 (PDB ID: 3LUT), EAG1 (PDB ID: 5K7L), TAX-4 (PDB ID: 5H3O) and TRPV1 (PDB ID: 5IRZ) voltage sensors showing important gating pore residues in stick representation.

Therefore, we decide to study the pattern of residue conservation in the gating pore both in representative V-dependent and V-independent channels, as well as its relationship with the overall flexibility of the VSD. As in K_V_1.2, the structure of K_V_10.1 shows that D264 (S2) and D299 (S3) potentially interact with R336 (S4) by forming salt bridges (3.3 and 4 Å respectively). On the other hand, in the CNG-like TAX-4 channel, the distance between D253 (S3) and R286 (S4) is 6.4 Å. In TRPV1 the only charged residues are E513 in S3 and R557 in S4, separated by a distance of 9.3 Å (*data not shown*). Neither of these cases is effective to form saline bridges and in consequence, those channels are virtually V-independent. We also compared the contribution of several gating pore residues, mainly located in S2, reported as important to facilitate the gating charge transfer in V-dependent channels and contrast them with the corresponding positions present in CNG and TRPV channels. In V-dependent channels, these acidic counter-charges and specific hydrophobic residues have been well identified by different groups [20,42]. In Figure 4 K_V_1.2 and K_V_10.1 were contrasted with TAX-4 and TRPV1 in order to evaluate how the CTC has evolved both in voltage- as well as in ligand-gated channels. Interestingly, residue pattern and the overall composition of specific residues are well conserved in K_V_ and CNG channels. In TAX-4, D218 (S2) and R286 (S4) could interact by salt bridges but weakly (distance = 4 Å). However, D208 (S2)/R280 (S4) and D212 (S2)/R283 (S4) have optimum distances to form salt bridges (3.4 and 3.6 Å, respectively). It has been proposed that R1–R4 movement in response to changes in membrane potential is facilitated also by F233 (F290 in *Shaker*) whose rigid ring appears to determine the main energetic barrier, enabling a switch-like transition between the closed-to-open state [62]. To do this, F233 exposes its aromatic side-chain to the center of the gating pore, forming an occluded site formed also by E236 and D259 (S3) [20]. Similarly, in K**_V_**10.1, F261 and D264 (S2) and D299 (S3) are almost disposed in the same configuration. This residue disposition is more or less conserved also in TAX-4; however, the kinked nature of S4 in this channel, due to high local flexibility at the C-end besides the presence of a short S4-S5L, makes TAX-4 sensitive only to a fraction of the transmembrane voltage field [52]. In TRPV1, on the contrary, the side-chain of the equivalent residue (Y487) is not oriented to the interior of the CTC (Figure 4).

Segment S3 of TRPV1 is rather more rigid and has only one Arg residue at position 557 (R6) in S4 which is too far to interact with E513 located at 8.5 Å in S3 (Figure 4 and *data not shown*). Likewise, the CTC in this family shows poor conservation with some members exhibiting small hydrophobic residues instead of the typical acidic side-chains used as counter-charges. This could indicate that V-dependence in some members of the TRPV subfamily has not been completely lost during the evolution of this family, consistently with previous reports [47]; however, the relevance of these observations deserves more investigation. Besides, molecular packaging of the VSD in TRPV1 is more constrained than in V-dependent channels, in part because the S3b–S4 *paddle* motif in K_V_ channels makes few critical contacts with the rest of the protein [63], which is also consistent with the local flexibility results presented here. Instead of voltage, TRPV1 is mainly activated by heat (> 42°C), pungent chemicals such as capsaicin, and by resiniferatoxin and vanillotoxins [53,64]. On the other hand, also in CNG channels, some detectable V-dependence has been described, particularly in the presence of large permeant cations [65]. Importantly, this peculiarity has been associated with the intrinsic pore flexibility reported in those channels [66]. In view of these findings, we decided to further explore if S3 flexibility is in some way correlated with V-dependence as a function of additional activators including diverse chemical compounds or “ligands”, the mechanical force exerted along the membrane, or the effect of temperature.

### Voltage- and ligand-dependence

As voltage dependence, ligand-dependent gating is widely distributed in the VGIC superfamily. The first impression of data plotted in Figure 1 indicates that both highly flexible channels (CNG, BK_Ca_ and SK/IK) (Figure 2 and Suppl. Figure 4) as well as proteins exhibiting noticeable rigidity (TRPV) are gated by some kind of ligand, although some subtle differences have to be underlined. Whereas CNG channels are almost completely dependent on cyclic nucleotides to be activated [67], TRPV channels are frequently considered as polymodal receptors responding to a plethora of stimuli, including a diverse spectrum of natural compounds with agonistic activity [9]. However, channels with high rigidity or intermediate flexible S3 profiles (K_V_7, K_V_10-12, Hv1) are typically gated only by voltage and just modulated by specific ligands such as divalent cations, protons and phosphoinositides [68–70]. On the other hand, channels exhibiting a highly flexible VSD, such as CNG or the Ca^2+^-activated BK_Ca_ channels (Slo or MaxiK channels), are sensitive to the membrane potential in a lesser extent than those channels with a more rigid profile, belonging to the K_V_ or EAG families, according to the reported elementary charges moving through the CTC during activation (Table 1). Furthermore, BK_Ca_ channels are not only directly activated by Ca^2+^ but they can be finely modulated by this cation in a dual mechanism of voltage/Ca^2+^ activation [71] and, besides Ca^2+^, activation can also be regulated by several ligands of diverse nature, including intracellular Mg^2+^, protons, CO, CH_3_OH, Heme, PIP_2_, Omega-3, temperature as well as several auxiliary β and γ subunits [10,72–74]. Thus, in general terms, S3 flexibility could be an attribute of ligand-dependent gating.

To assess this hypothesis, close inspection of the Slo1 (BK_Ca_) structure allows establishing a correlation between the intrinsic flexibility of S3 and its mechanism of gating. Whereas in *Shaker*-like K_V_ channels, segment S4 is separated from S5 by a long linker (S4-S5L), the S4 helix in BK_Ca_ channels is tightly packed against it by a very short connector. In consequence, *Shaker*-like channels apparently undergo much larger voltage-dependent conformational changes than BK_Ca_ channels [20,25]. According to our analysis, although the S3 flexibility profile in members of the K_V_1-4 families is slightly lower than the one found in BK_Ca_ channels (Figure 1 lower panel), it significantly increases if segments S3-S4 or the whole VSD is considered (Figure 1 *middle* and *upper* panels). This indicates that both *Shaker*-like and EAG (K_V_10-12) channels are, overall, more flexible than BK_Ca_ channels, which in this case is not consistent with our assumption linking a flexible VSD with an increasing dependence on ligand binding. However, the high flexibility observed in the V-sensors from K_V_1-4 and K_V_10-12 channels should be mainly attributed to the presence of long linkers (S1-S2L, S2-S3L, S3-S4L, S4-S5L) instead of the short ones present in the BK_Ca_ family [51,75,76]. In addition, if the domain-swapped architecture of K_V_1-4 channels for the VSD and the deep dependence on voltage exhibited by such proteins is considered, it can be more easily explained how these channels undergo large conformational changes in comparison with BK_Ca_ channels. From this perspective, the slow rearrangements previously reported for the S3-S4L in BK_Ca_ channels [77] and the fast rearrangements for the same segment in K_V_1.2 [78] can be better understand as a function of their specific local flexibility.

### Voltage- and temperature-dependence

**Figure 5.**
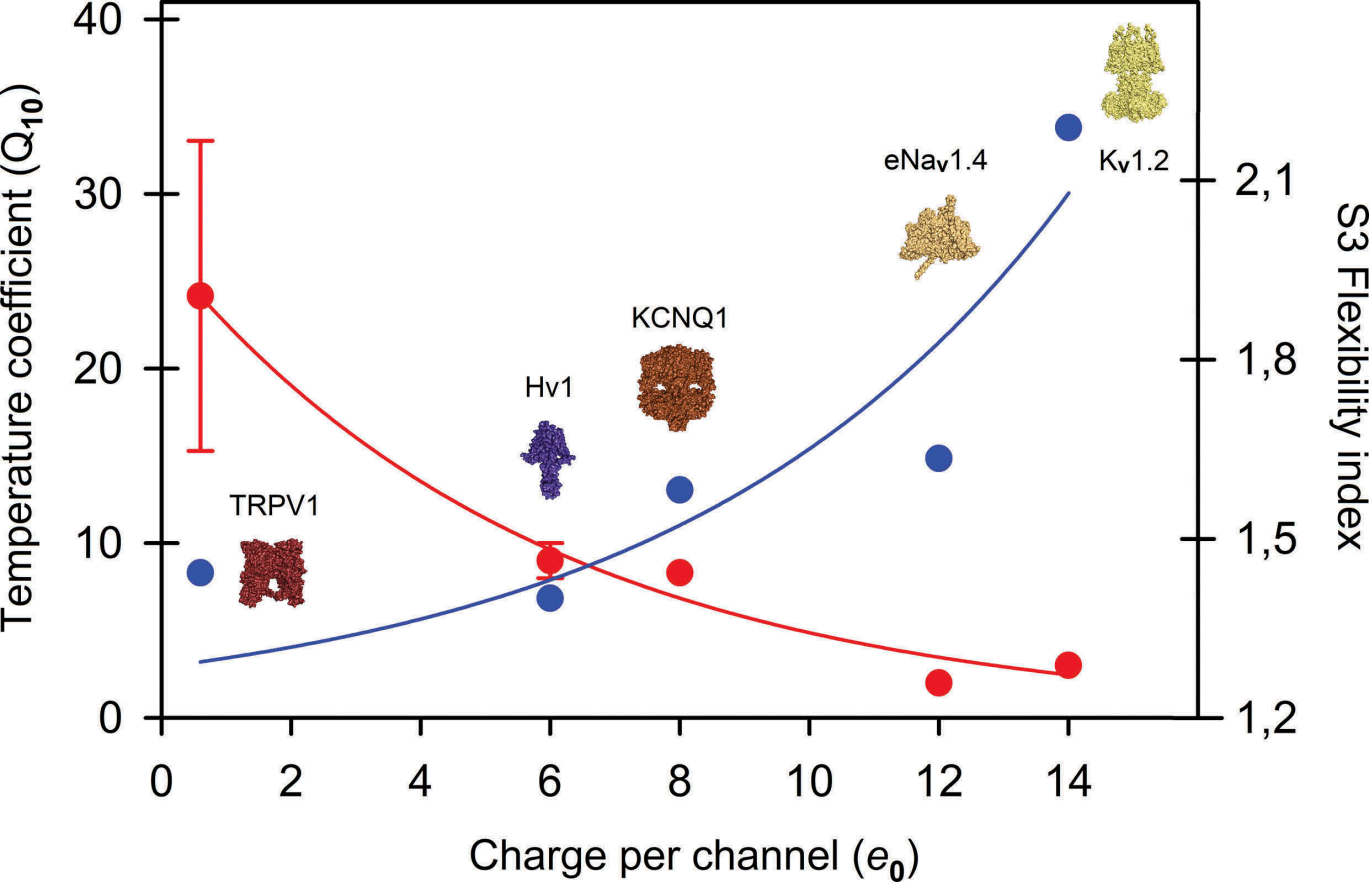
Activation parameters for specific members of the VGIC Superfamily and their relationship with S3 flexibility measured as the mean B-factor. The S3 flexibility (*blue* circles) and the coefficient of temperature for activation (*red* circles) are plotted as a function of the voltage dependence of the channel, measured as the number of charges participating during activation. Structures of ion channels are shown as surface projections in increasing order of flexibility for the S3 segment. Best fitting for Q_10_ (*red* curve) or mean B-factor (*blue* curve) as a function of the number of charges *per* channel is shown. Flexibility data for sodium channel corresponds to the Na_V_1.4-DI from *Rattus novergicus* whereas the structure corresponds to the Na_V_1.4 channel from *Electrophorus electricus* (PDB: 5XSY)

Our next aim was to evaluate if activation by temperature is in some way influenced by local flexibility of the V-sensor, and then determine if there is a dependence on this physical variable in relation with the sensitivity to voltage. Hence, we evaluated the reported Q_10_ temperature coefficients of specific gating parameters for members of the VGIC superfamily. Since the time course of the increase of current during a voltage step reflects the speed with which the channel opens, we compared the time constant of activation (τ_act_) and the one for inactivation (τ_inact_) or deactivation (τ_deact_) as a measurement of the kinetics of the channel during gating. Table 1 also summarizes the temperature dependence of these kinetic parameters. We found that high S3 local rigidity found in TRPV, Hv1, K_V_7 and the domain DIV of Na_V_1 channels correlate well with a strong dependence on temperature and *vice versa*. Specifically, TRPV channels, whose S3 and S3-S4 profiles are one of the most rigid into the VGIC superfamily, are indeed considered as thermoreceptors, predominantly activated by heat (> 42°C, TRPV1, Q_10_ = 15 − 40) or noxious heat (> 52°C, TRPV2, Q_10_ > 100) [79]. The proton channel Hv1 exhibits even a more rigid profile for the equivalent segment. In this case, a strong temperature dependence (Q_10_ ≈ 9) for several gating parameters has also been demonstrated. This coefficient is considered big in comparison with other V-dependent ion channels with nominal temperature dependence (Q_10_ < 3) [80]. Only K_V_7 channels, which are more rigid than *Shaker*-like and EAG (K_V_10-12) channels, are considerably more dependent on temperature (Q_10_ ≈ 8) [81]. Notably, in Na_V_ channels, the main component for fast inactivation, the rigid domain DIV, also show more dependence on temperature in comparison with the two main components involved in activation, i.e. the DI and DII domains [82], which are significantly more flexible (Figure 1). In Figure 5 the complex relationship between V- and T-dependence is shown as a function of the S3 flexibility for these specific ion channels.

### Voltage dependence and mechanosensitivity

Our last aim was to explore channel activation triggered through membrane stretching, and again try to shed some light on the significance of the specific local flexibility on this mechanism. During conformational changes underlying the opening and closing activity of voltage-gated channels, the motion of VSDs interacting with adjacent lipids is required. This renders channel activity energetically sensitive to the bilayer’s mechanical state [83]. Under this theoretical framework, it should not be surprising that ion channels are intrinsically mechanosensitive. Thus, there is considerable evidence regarding the fact that membrane stretch is responsible to modulate the activation of voltage-, ligand- and Ca^2+^-gated channels [84,85]. Indeed, the so-called force-from-lipid principle is so basic that it can be applied to a plethora of biomolecules, particularly to membrane-embedded proteins, from the beginning of cellular evolution [86]. However, albeit mechanosensitivity might be an attribute of all membrane proteins, in comparison with true mechanosensitive channels such as MscL, MscS, or TRAAK and TREK1 channels, voltage-gated channels are less sensitive to the effects of membrane tension. In the K**_V_**1.2 *paddle* chimera, for example, sensitivity to lateral tension is approximately one-tenth the one required to open the mechanosensitive channel of large conductance, MscL [87]. On the other hand, analysis of the structure and gating of MscS indicate that tension- and voltage-sensitivity could be coupled so that, as the membrane is depolarized, less tension is required to open the channel and *vice versa* [88,89]. These interesting relationships led us to compare the S3 flexibility in specific members of the VGIC superfamily with the evidence reported on mechanosensitivity by membrane stretching in those channels.

In MscL and MscS, a common NxxD motif actively participate in important conformational changes leading to the opening of the nanopore. In MscL, this motif serves as a hinge to stabilize the open state through the N-terminal S1 helix [90] while in MscS, the conformational state of the channel depends on the helical propensity of TM3 provided by the flexibilities of Gly113 (for inactivation) and Gly121 (for desensitization) [91]. Notably, Gly121 is best conserved than Gly113, being aspartic acid more common in that position [92], which is the same residue present in the NxxD motif. The point is that in both cases this sequence motif is intrinsically flexible, and it contributes to the bending of the TM1 and TM3 helices respectively (*see* Suppl. Figure 1 and the companion article). With this in mind, we decided to include the flexibility profile of the TM1 segment of MscL in our analysis and we found that it belongs to the more flexible segments studied here. However, if the entire TM1-TM2 segment is considered, a much more rigid profile emerges, comparable to the one present in domain DIV from Na_V_1 channels (Figure 1 *middle* panel). This is consistent with the presumed link between segmental rigidity and mechanosensitivity. On the contrary, the S3-S4 flexibility in CNG channels reaches a maximum in the VGIC superfamily, which could suggest that, since they have evolved to be mainly activated by cyclic nucleotides instead membrane stretching, a flexible profile of the V-sensor is disadvantageous for mechanosensitivity. Indeed, there are no reports on the literature of CNG channels activated by lateral tension, even considering that these proteins show some sequence similarity to the bacterial mechanosensitive channel of small conductance, MscS [93]. In a similar context, there are some reports suggesting that TRPM and TRPA channels, whose S3 segments are less flexible, could are in some way mechanosensitive [94]. In the case of BK_Ca_ channels, also exhibiting a lesser flexible S3 profile in comparison with CNG channels, currents are practically not affected by osmotic challenges and are mainly insensitive to the stretching of the membrane [95, although mechanosensitivity depends on the experimental conditions [95]. Rather, as we pointed out before for temperature dependence, we found that modulation of activation by mechanical stretching in the VGIC superfamily is preferably associated with rigid S3 profiles [95].

The best way to quantify this property is by comparing the midpoint of activation shift (*Δ*V_1/2_) as a function of the membrane stretching (Table 2). Some of the S3 profiles with more pronounced rigidity are members of the TRPV, Hv1, and KCNQ families, as well as domains DIII and DIV of Na_V_1 channels (Figure 1). In accordance with our prediction on S3 rigidity facilitating mechanosensitivity, KCNQ channels, TRPV2 and Hv1 have recently been recognized as directly activated by the membrane stretching [95–98]. In Hv1 proton channels, for which the whole VSD is the more rigid in the VGIC superfamily (*see* Figure 1, *top* panel), the effect of negative pressure of – 10 mmHg in the pipette clearly shifts the activation curve ∼14.5 mV [98]. Mechanosensitivity has also been reported in Na_V_ and Ca_V_ channels, and we detected the same pattern. The S3 segments from domains DIII and DIV of Na_V_ channels are comparatively more rigid than the analogous segments in domains DI and DII (Figure 1, Suppl. Figure 5). Interestingly, the DIII-DIV linker is critical for fast inactivation in Na_V_ channels and, indeed, it has been determined that movement of the V-sensor in domain DIV is the rate-limiting step for this process while the motion of the corresponding sensor in DI is the rate-limiting step during activation [48]. According to previous reports [99,100], membrane stretching reversibly accelerate both activation and inactivation in a similar way.

**Table 2.** S3 Flexibility and stretch-induced shifts of activation.

Channel	S3 Flexibility index (1/mBf)	S3-S4 Flexibility index (1/mBf)	Charge per channel (*e*_0_)	Mechanosensitivity *V*_1/2_ shift (Δ*V*_1/2_, mV)	References
Na_v_1.5 DI^a^	1.61	1.71	~ 12	~ – 5 to – 35^b^	[99,100]
BK_Ca_ (Slo1)	1.55	1.67	2.4–4.4	~ – 4 to – 20 **^c^**	[95,146,147]
Na_v_1.5 DIV	1.55	1.64	~ 12	~ – 5 to – 35^b^	[99,100]
*Shaker* K_v_	1.48	1.96	12 – 16	–8.8 **^±^** 1.3	[148]
Ca_v_ 1 DI^d^	1.48	1.79	~ 9	–1.4 **^±^** 0.4	[149,150]
Na_v_1.5 DIII	1.45	1.73	~ 12	~ – 5 to – 35^b^	[99,100]
Ca_v_ 2 DIII^e^	1.44	1.92	~ 9	~ 0^f^ ; –7 to – 18 ^g^	[104]
K_v_7.1 (KCNQ1)^h^	1.41	1.61	~ 8	–13 **^±^** 1.9 **^i^**	[96]
hHv1	1.40	1.49	6	–14.4	[98]

Notes for Table 2

**^a^**Human sodium voltage-gated channel type 5 from cardiac muscle (Uniprot: Q14524)

**^b^**Voltage dependence of activation at pressures −10 mmHg to −50 mmHg

**^c^**Δ*V*_1/2_ depends on the experimental conditions and expression system but it is always more pronounced at very depolarized voltages

**^d^**Ca_V_1.2, Acc.: P22002. Expressed in heart, pituitary, adrenal gland, liver, kidney, and in a much lesser extent in testes and spleen

**^e^**Ca_V_2.2, Acc.: Q00975. Human N-type brain calcium channel, isoform 5

**^f^**Activation *V***_1/2_** values and *k* (slope factor) showed no significant changes with time

**^g^**Inactivation relationships obtained just after whole-cell access or with no stretch just after access respectively

**^h^**Human potassium voltage-gated channel subfamily KQT member 1 (Uniprot: P51787)

**^i^**An increase in current, acceleration of activation, and slowing of deactivation under stretching is also observed

In calcium channels, an equivalent but more subtle relation was also found. Similarly, a different study shows that Ca_V_ channels have at least two separate regions of the α-subunit which are involved in fast inactivation, namely the linker between domain DI and DII, and the S6 regions in domains DII and DIII [101,102]. Contrarily to the situation in Na_V_ channels, segment S3 in domains DI and DII in Ca_V_ channels is comparatively more rigid than the one present in domains DIII and DIV (Figure 1, Suppl Fig. 6), which are mainly involved in channel activation [103]. Remarkably, in Ca_V_2 channels, it has been reported that steady-state activation curves show no sensitivity to the stretching of the membrane whereas inactivation curves exhibit ∼18 mV leftward shift when the membrane is stretched [104] (Table 2). Taken together, these findings suggest that in addition to specific structural factors, conformational changes associated to fast inactivation in Na**_V_** and Ca_V_ channels could be linked to some degree of local rigidity of a specific segment of the protein, whereas activation requires more flexible segments of the V-sensor. In any case, more research should be conducted to explore these hypotheses.

## Discussion

Flexibility is critical for protein structure, stability, and function. In this study, sequence analysis and prediction of local flexibility have revealed that two important triggers/modulators of gating in VGICs, seems to be directly associated with the intrinsic flexibility of the S3 segment: temperature and membrane stretching. On the other hand, we have shown that voltage- and ligand-dependence is widely distributed in the vast majority of members of this superfamily. In addition, we also show that both in channels with high flexibility profiles (CNG, Ca_V_ DIV, K_V_1-4, TRPM) and in those with significant rigidity (TRPV, Na_V_ DIII and DIV, K_V_7, and Hv1), the dependency on voltage or ligand seems not to be associated with the local flexibility exhibited by segment S3, the one for the S3-S4 hairpin or the whole VSD. Therefore, our analysis highlights the relevance of local flexibility in determining how VGICs respond to temperature and the membrane stretching, and how this intrinsic property is specifically adapted both in V-dependent and ligand-gated channels. In future studies, these questions might be experimentally addressed. The present study also has exposed the degree of conservation in specific elements that this vast group of proteins exhibit. Furthermore, we have focused on three structural aspects of these proteins, relevant for gating: (1) the so-called *paddle* motif; (2) the charge transfer center, and (3) the global VSD architecture as a function of the degree of local flexibility in specific segments. Taking advantage of the available structural data for the most representative members of this superfamily, it is hypothesized that local flexibility could have an important impact on several aspects concerning ion channel gating. Nevertheless, every case should be analyzed in detail in future studies.

The main aspect linked to S3 flexibility is thermal activation. Our estimates strongly suggest that this parameter and conformational S3 flexibility are closely correlated. We found that some degree of rigidity favor activation by high temperatures; this is mainly the case of thermosensitive TRPV and Hv1 proton channels whose amino acid preferences and distribution determine a rigid profile for S3 or S3-L-S4 segments. Indeed, the whole VSD of proton channels is compositionally more rigid than the one present in anyone member of the VGIC superfamily (Figure 1). Proton channels exhibit one of the higher Q_10_ values for the *tau* of activation, just below the heat activation exhibited by thermo-TRP channels TRPV1 and TRPV2 (Table 1). In ion channel thermodynamics, a high value of Q_10_ is indicative of the existence of a large enthalpic component over a short temporal period during the closed-to-open transition [105]. Such a component is important to the adjustment of conformational flexibility during the thermal adaptation of proteins [13]. Interestingly, the TRPV1 channel gating is also accompanied by large entropic and enthalpic changes, which suggest substantial conformation changes in specific zones of the protein [106]. Consistent with this, structural evidence indicates that the `V-sensor´ of TRPV1 is  virtually static during the closed-to-open transition [28,53], but some reports indicate a high dynamism of the pore and the S4-S5L in those channels [29,56,58,59]. On the other hand, channels considered flexible (as the members of the *Shaker*-like K_V_ family) or extremely flexible (such as CNGA2) at the S3 segment, exhibit weak dependence on temperature with Q_10_ values below 4 [107,108]. Notably, the mean Q_10_ in CNGA2 can reach a maximum of ~ 6 only if [cGMP]/EC_50_ = 100nM [108]. This suggests that, in addition to intrinsic flexibility, ligand-binding synergistically contributes to thermal activation on these flexible channels. One thermodynamic implication of this could be that flexible channels are probably better adapted to be activated under cooling. The best example is the cold receptor TRPM8, which is significantly more flexible than TRPV1 but less than CNGA2 (Figure 1). In this case, the overall architecture of the channel as well as its energetic landscape, strongly determines its dependence on cooling for activation [47]. Similarly, important conformational changes upon temperature activation have also been reported for this channel [106]. However, the transmembrane S1-S4 domain of TRPM8, in contrast to the one present in TRPV1, is more dynamic and capable to accommodate chemically different agonists [60]. Thus, our results show a subtle and probably unexpected link between local flexibility, thermal activation and ligand-dependence that deserves more investigation.

The fact that local flexibility/rigidity determines the thermal dependence of an ion channel to be activated, reveal the well-known thermoadaptation in thermophilic proteins. Although each case must be experimentally addressed in detail, in general terms theory predicts that an increase in the mechanical rigidity of specific protein segments facilitates thermal adaptation by allowing those regions to move as rigid bodies with defined degrees of freedom, motion facilitated under high temperatures. Indeed, in rigid regions, atomic fluctuations and side-chain torsion are more restricted [109], which means that the local substructure could be better stabilized at high temperatures. In this situation, a decrease in temperature should reduce even more these degrees of freedom, leading to losing the function. On the other hand, high flexibility is required to achieve small-scale motions, facilitating deformations under cooling conditions, more difficult to be detected [110]. Hence, a flexible profile could be better adapted to reach effective degrees of freedom at low temperatures due to an increase in the stability of the structure. However, if these regions are exposed to high temperatures, too many degrees of freedom could be disadvantageous leading to the destabilization, unfolding and losing the function of the protein.

As proteins are compositionally heterogeneous, rigid-to-flexible profiles are scattered throughout the primary sequence. Moreover, protein folding, and the solvent accessibility issue increase structural complexity and difficult the proper interpretation of protein function [40]. In order to solve this, identification of the nonhomogeneous distribution of flexibility profiles is pivotal, besides such information can take advantage of structural data to correlate local flexibility with a specific motion. Hence, the use of state-of-art experimental approaches, such as NMR, single-molecule force spectroscopy, or circular dichroism allows to further explore, at atomic detail, the conformational flexibility, molecular dynamics, and ligand interactions in specific conditions. With these considerations, local flexibility predictions should also be contrasted with experimental data which deal with no purely conformational phenomena but the effect of extrinsic factors such as glycosylation, saline conditions, pressure, solvent viscoelasticity, and density issues. Furthermore, side-chain flexibility reflects an intrinsic property of lateral chains which is correlated to configurational entropy differences [39,40]. Therefore, the internal motion also depends on several factors including heat capacity, salt bridge networks, electrostatic and dipole interactions, as well as the hydrophobic effect [14]. Notably, in addition to the important effect that local flexibility could have on the VSD motion, ion channel gating also could depend on the resulting state-dependent changes in solvation of specific microdomains distributed over the whole channel [111].

Last but not least, our results clearly indicate a connection between sensitivity to membrane stretching and the relative rigidity of the S3 segment. This is more evident in TRPV, Hv1 and K_V_7 channels. Why does a rigid S3 profile apparently favor the mechanosensitivity of a V-gated ion channel? Flexibility in proteins has been categorized as: (1) *systemic* referring to small-scale fluctuations in side- and main-chain atoms in their native states, and (2) *segmentary* if the motion of one part of the protein with respect to one another is triggered in response to a specific molecular event [112]. According to this logic, protein movements due to segmental mobility are much larger than the ones due to systemic flexibility. In this context, segmental flexibility in proteins is mostly restricted to the motion of specific subdomains and is frequently associated with the presence of molecular hinges moving big rigid bodies. This is the case of the S6 hinge facilitating the motion of the lower gate in *Shaker*-like channels [1,25]. Thus, in order to better respond to the lateral tension, segmental motion of big rigid bodies could be advantageous to adapt the protein to the deformation of the lipid bilayer under such condition. However, we found that MscL mechanosensitive channels are compositionally flexible for TM1 segment (Suppl. Figure 1). This could suggest that other important parameter to consider is the architecture of the channel, which in the case of MscL allows an easy transition to the open state under conditions of high membrane tension, rotating and tilting the TM domains as big rigid bodies [113]. This transition is highly dynamic and mainly determined by protein/lipid interactions, lateral tension of the bilayer, TM helix tilting, substantial rearrangements in both TM1 and TM2, lipid adaptation, and membrane thinning, resulting in channel expansion [114]. As the architecture of VGICs is completely different, the motion of specific rigid bodies could be preferred since in this way the effect of stretching should be better transmitted to stabilize the open state. On the contrary, a soft body, more deformable, could be disadvantageous, mitigating the effect of lipid adaptation under conditions of high lateral tension. Therefore, systemic flexibility, mainly associated to flexible segments, could be restricted with more modest conformational changes, as the ones detected in the S4 region of BK_Ca_ channels [77]. The implications of these arguments are open questions to be experimentally addressed in the future.

Experimental and theoretical evidence confirm that some of the most rigid channels studied here (*i.e*. TRPV1,2,4, Hv1, K_V_7) are implicated in major structural rearrangements during gating [28,58,59,115,116]. In Na_V_1.5, whose S3 segment is slightly more rigid than the one in domains DIII and DIV of Ca_V_1.1 channel, Beyder et al. have reported a marked leftward shift both for activation and inactivation in response to membrane stretching [100]. In Ca_V_1.1, which is significantly more flexible for these segments, Calabrese et al. have reported that only inactivation is mechanosensitive [104]. In both channels, fast inactivation is associated to a particle – the *ball* – that physically occludes the pore by interacting with other domains and which is part of an intracellular linker situated between domains DIII-DIV in Na_V_ channels [49] or DI-DII in Ca_V_ channels [101,102], both of them rigid. This evidence is consistent with the concept of segmental rigidity participating in large-scale molecular motions.

According to our analysis, a physical correlation between S3 rigidity, sensitivity to membrane stretching and fast inactivation in sodium and calcium channels is proposed. We hypothesized that segmental rigidity is associated with large-scale motions of specific domains during inactivation as well as systemic flexibility and small-scale molecular motions are preferentially involved during activation in specific regions of the V-sensor. In favor of this, some reports indicate that, in flexible channels (BK_Ca_, SK, CNG) gating is not mainly mediated by the large bending of the distal part of S6 (such as in K_V_, Na_V_, or Ca_V_ channels) but small conformational rearrangements in the selectivity filter and the S4-S5/S5-P-helix connecting loops, which play a major role on this process [30,117,118]. Obtaining this type of evidence will help to corroborate or rule out our conjectures. In any case, prediction of local flexibility, molecular dynamics, and the adequate use of experimental tools could help to answer many of these questions.

## Concluding remarks

By exploring and predicting local flexibilities in several members of the VGIC superfamily, we show that – in general terms – while the voltage- and ligand-dependence do not seem to be related with the V-sensor’s flexibility, temperature and membrane stretching dependences are more related with this parameter, particularly if S3 segment is considered. Hence, voltage dependence is widely distributed in the VGICs superfamily but the S3 flexibility is not necessarily linked to this attribute since it is presented both in channels with flexible and rigid S3 segments. However, channels with highly flexible profiles (as CNG) or with significant rigidity (as TRPV) are essentially independent of voltage. In any case, protein architecture, more than amino acid composition, strongly determine the V-dependence of an ion channel. S3 flexibility is mainly determined by two sequence motifs encompassing 12 residues and situated at the N-terminal part (the NxxD motif) and at the middle part of S3 (the start of the so-called *paddle* motif). In general terms, the S3 flexibility importantly contributes to the overall flexibility of the V-sensor (see Scheme). Hence, we have detected channels with high, intermediate, and low S3 flexibility profiles which are correlated with the effect that temperature or mechanical stretching exert on activation.

Our results also demonstrate that the approach of using compositional analysis and evaluation of normalized B-factors of individual amino acids is a trustworthy predictive procedure that allows interpreting local flexibility in structural terms. Nevertheless, flexibility is distributed not homogeneously throughout the V-sensors in the superfamily; therefore, flexibility data should be analyzed individually by considering the local environment at the atomic scale and should be contrast them with the available structural data. To do this, H-bonding, electrostatic interactions, hydrophobicity, synergistic effects between side-chain rotamer conformations, as well as the contribution of a delicate balance of entropic and enthalpic components should be investigated. Local flexibility prediction can shed some light on the fine-tuning between all these factors. This constitutes one of the challenges for the full understanding of the molecular mechanisms involved in ion channel gating.

## Note added in proof

The findings of Zubcevic and Lee [119] and Chen et al. [120], in light of the results discussed in this paper, suggest that rigid voltage sensors (*v. gr*. TRPV family) are more suitable for α-to-π helical transitions, while those that are more flexible (*v. gr. Shaker*) prefer α-to-3**_10_** transitions.

**Scheme.**
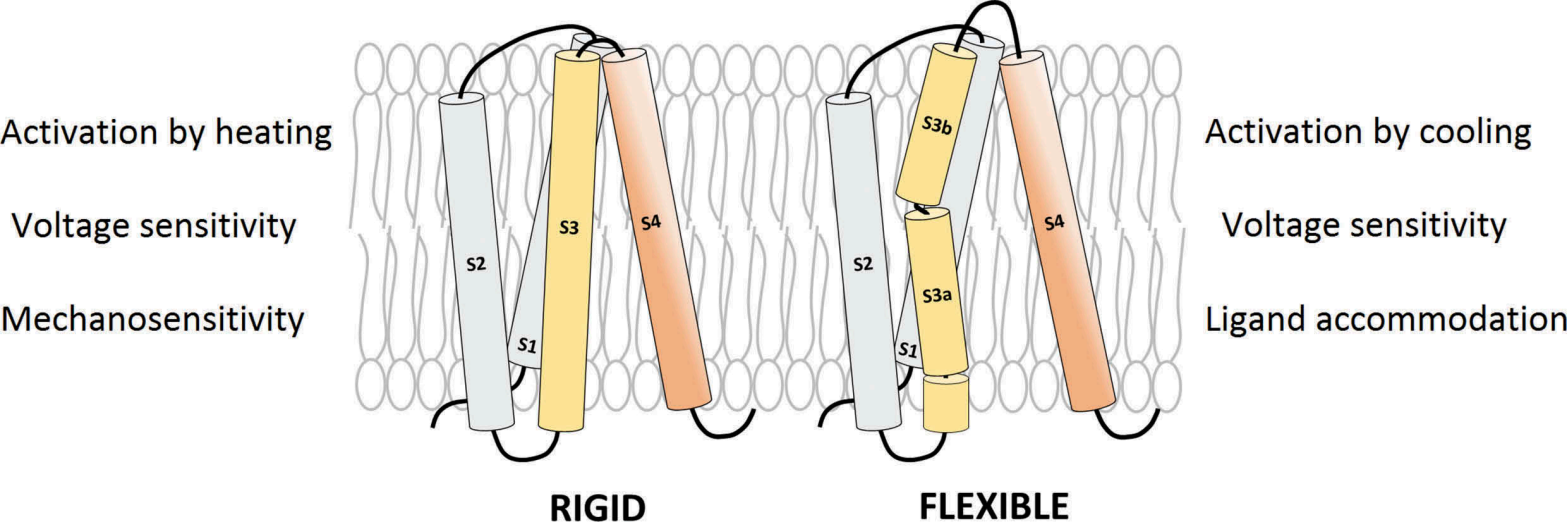
The voltage-sensor can be rigid or flexible, mainly due to the composition of S3 segment and the length of the connecting linkers. This has important conformational implications mainly in response to changes in temperature or the membrane stretching. Voltage and ligand dependences are widely distributed into the VGIC superfamily.
