# Supplementary material for: Voltage vs. Ligand I: Structural basis of the intrinsic flexibility of S3 segment and its significance in ion channel activation
Source: Channels (Austin). 2019 Oct 24;13(1):455–76. doi: 10.1080/19336950.2019.1674242 (PMC6833973; doi:10.1080/19336950.2019.1674242)
Supplement: Supplemental Material [file kchl-13-01-1674242-s001.zip › Supplementary caption.docx]

**Supplementary Fig. 1.** Predicted TM1 flexibility profile for the large-conductance mechanosensitive ion channel (MscL) family. Arrows denote the location of the NxxD and the conserved Gly 24 (TbMscL numbering). The structures of MscL in the closed state from *Mycobacterium tumefaciens* (PDB: 2OAR) and intermediate expanded state from *Staphyloccocus aureus* (3HZQ) are shown.

**Supplementary Fig. 2.** Predicted S3 flexibility profile for the K_V_1-4 and K_V_7 families. Arrows denote the location of the NxxD and *paddle*-motifs. The S3-S4 structure of K_V_1.2 from *R. norvergicus* (3LUT) in the closed state is contrasted with the S3-S4 structure of KCNQ-type K_V_7.1 from *Xenopus laevis* (5VMS). Inset shows atomic distances between K306 and R309 (S4) with D259 (S3).

**Supplementary Fig. 3.** Predicted S3 flexibility profile for the ether-à-go-go (Eag, K_V_10-12) and K_V_7 families. Arrows denote the location of the NxxD and *paddle*-motifs. The S3-S4 structure of K_V_10.1 from *R. norvergicus* (5K7L) in the closed state is contrasted with the S3-S4 structure of K_V_11.1 from *Homo* in the open state (5VA1).

**Supplementary Fig. 4.** Predicted S3 flexibility profile for calcium-activated potassium channels (K_Ca_). Arrows denote the location of the NxxD and *paddle*-motifs. The S3-S4 structures of K_Ca_1.1 (aSlo1) from *Aplysia californica* in the closed (5TJI) and open states (5TJ6) are contrasted with the S3-S4 structures of K_Ca_3.1 (hSK4) from *Homo* in the closed (6CNM) and open state (6CNO).

**Supplementary Fig. 5.** Predicted S3 flexibility profile for the Na_V_1 family. Arrows denote the location of the NxxD and equivalent *paddle*-motifs. The S3-S4 structure of eNa_V_1.4 from *Electrophorus electricus* (5XSY) in the closed state is shown contrasting domains DI and DIV.

**Supplementary Fig. 6.** Predicted S3 flexibility profile for the Ca_V_1 family. Arrows denote the location of the NxxD and equivalent *paddle*-motifs. The S3-S4 structure of Ca_V_1.1 from *Oryctolagus cuniculus* (rabbit) (6BYO) in the inactivated state is shown contrasting domains DII and DIV.

**Supplementary Table 1.** Sequence composition, conservation and mean flexibility for the segment S3 in the VGIC superfamily.
