# Supplementary figures and images for: Voltage vs. Ligand I: Structural basis of the intrinsic flexibility of S3 segment and its significance in ion channel activation
Source: Channels (Austin). 2019 Oct 24;13(1):455–76. doi: 10.1080/19336950.2019.1674242 (PMC6833973; doi:10.1080/19336950.2019.1674242)

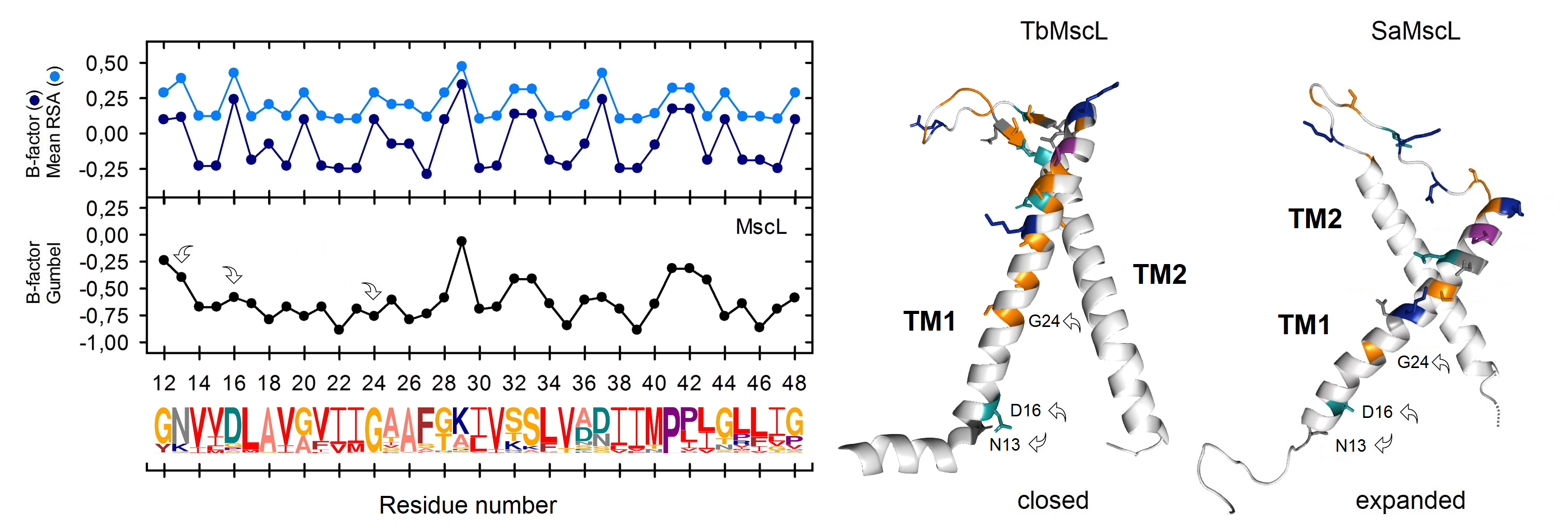

Supplement: Supplemental Material [file kchl-13-01-1674242-s001.zip › Supp-F1_MscL.png]

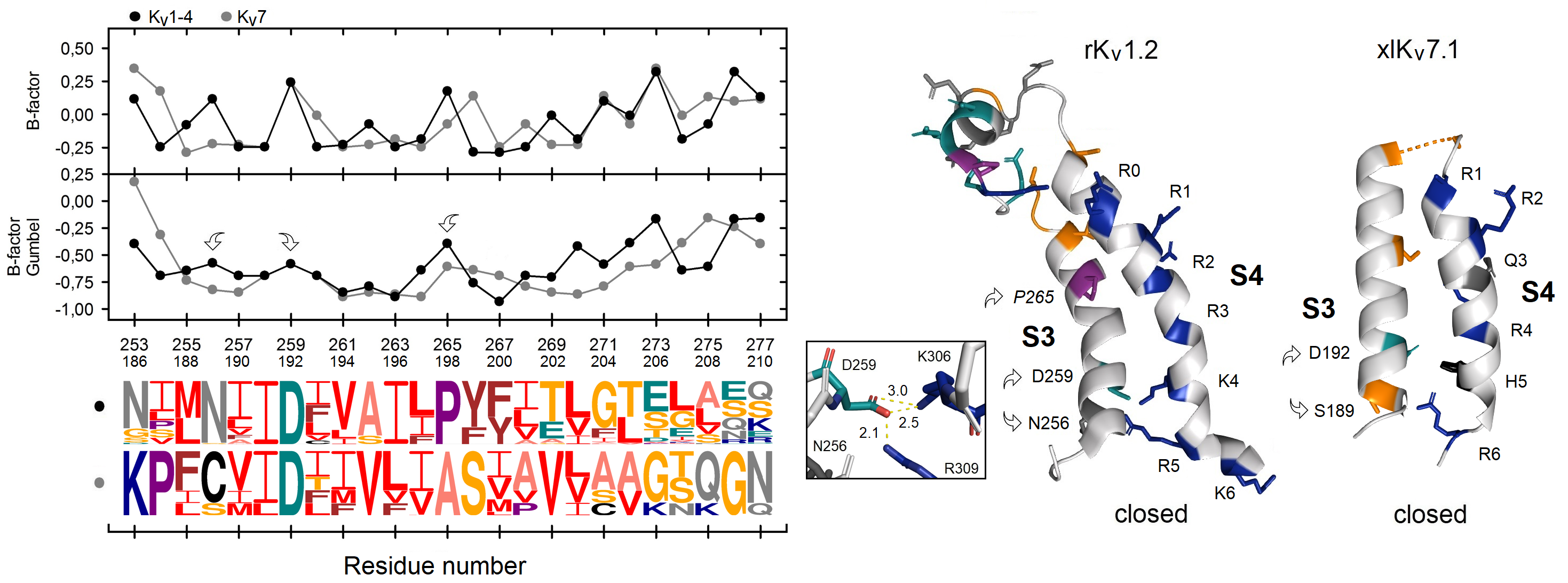

Supplement: Supplemental Material [file kchl-13-01-1674242-s001.zip › Supp-F2'_Kv1-4-7.png]

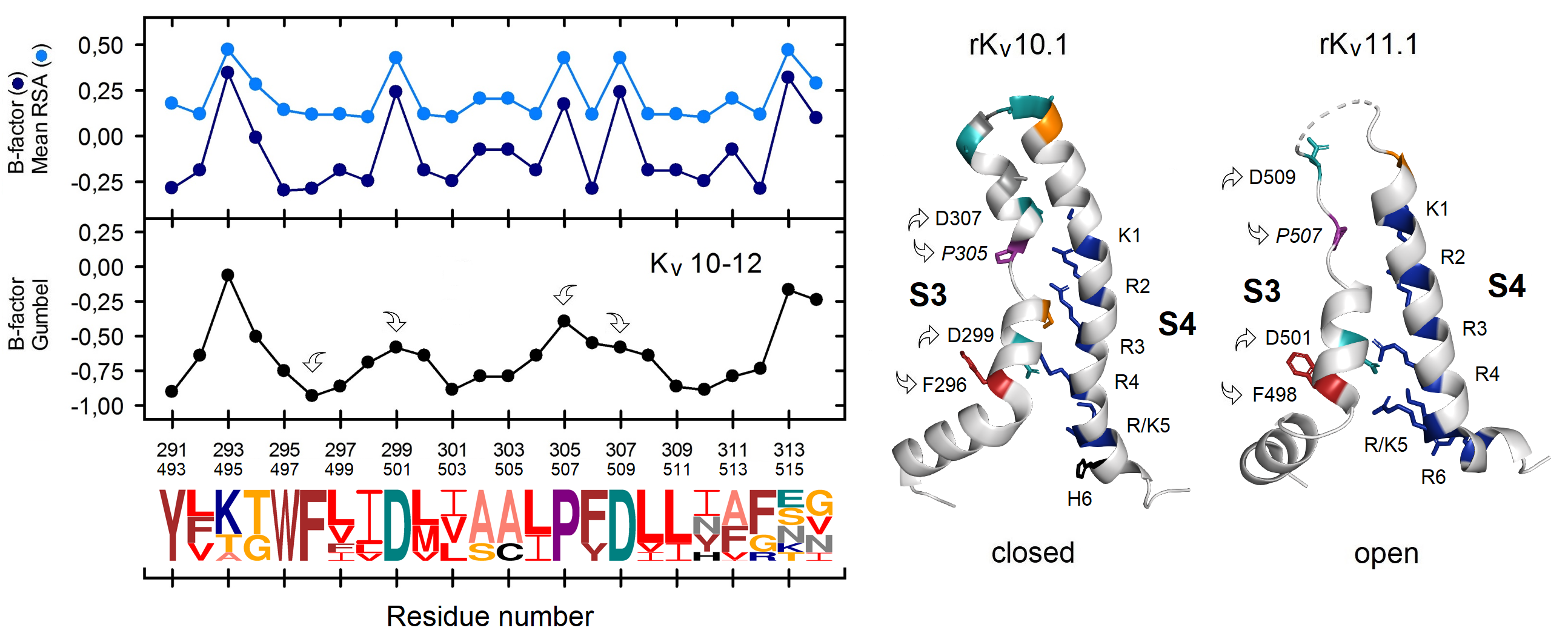

Supplement: Supplemental Material [file kchl-13-01-1674242-s001.zip › Supp-F3_EAG.png]

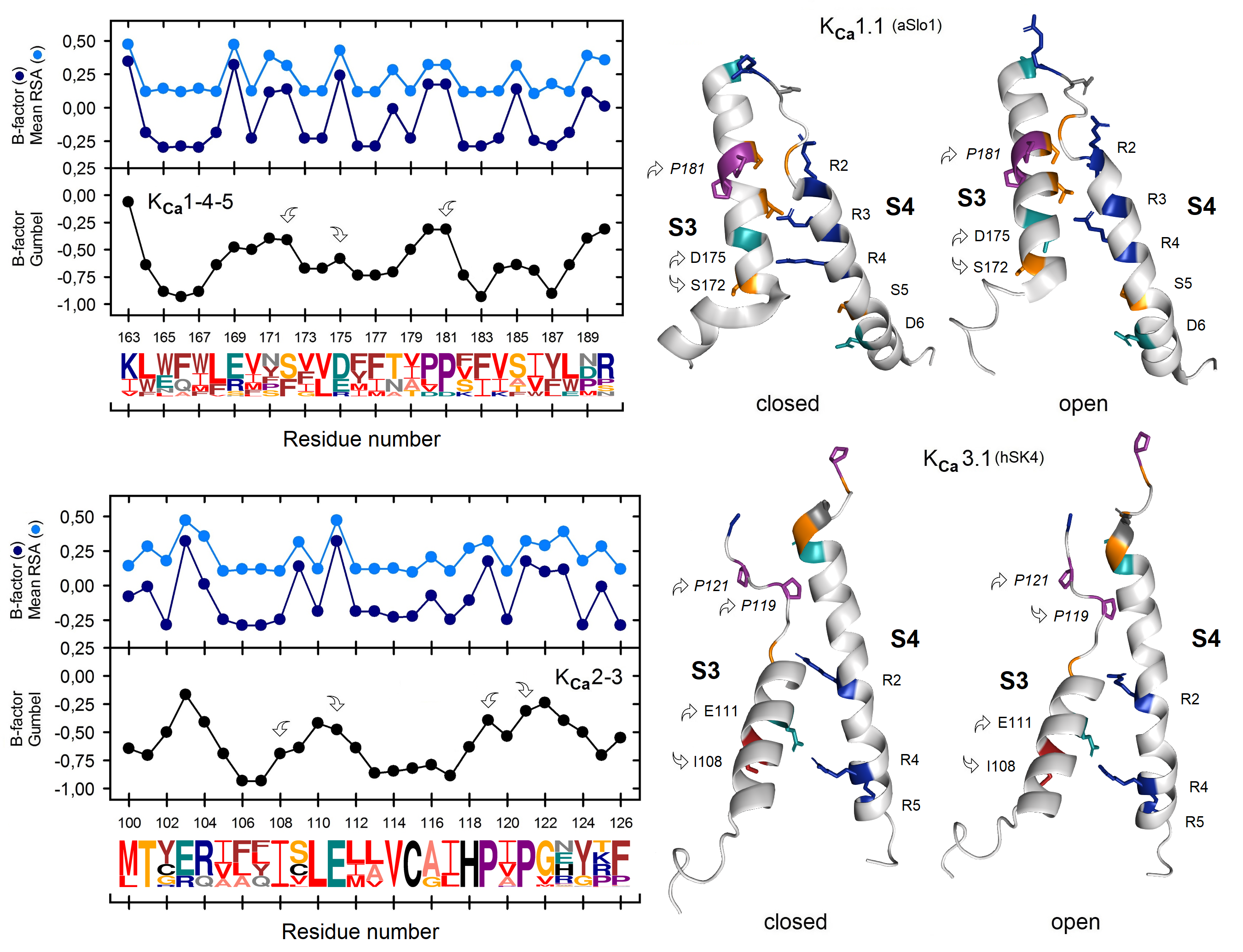

Supplement: Supplemental Material [file kchl-13-01-1674242-s001.zip › Supp-F4'_BK-SK.png]

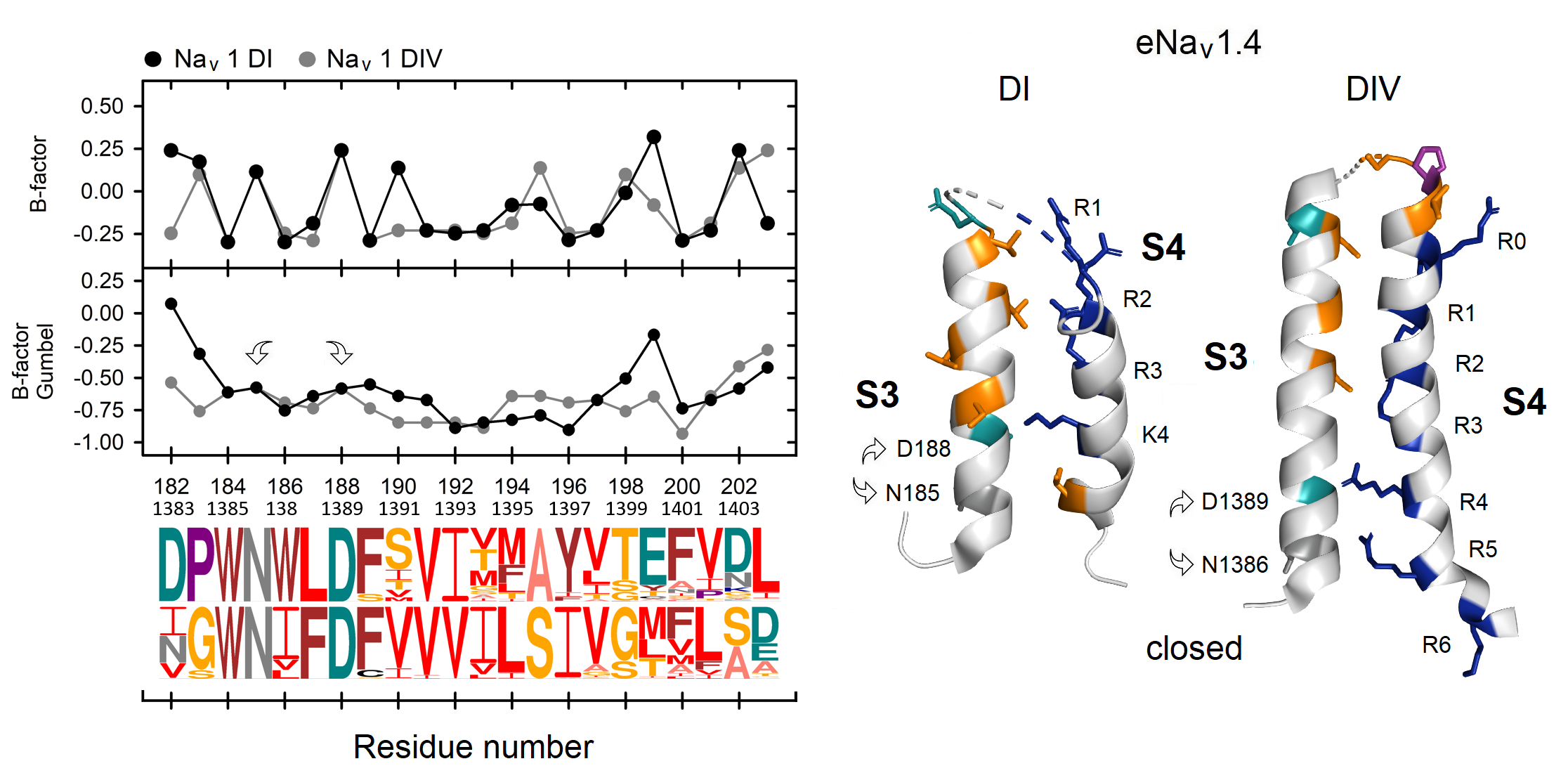

Supplement: Supplemental Material [file kchl-13-01-1674242-s001.zip › Supp-F5'_Nav1.png]

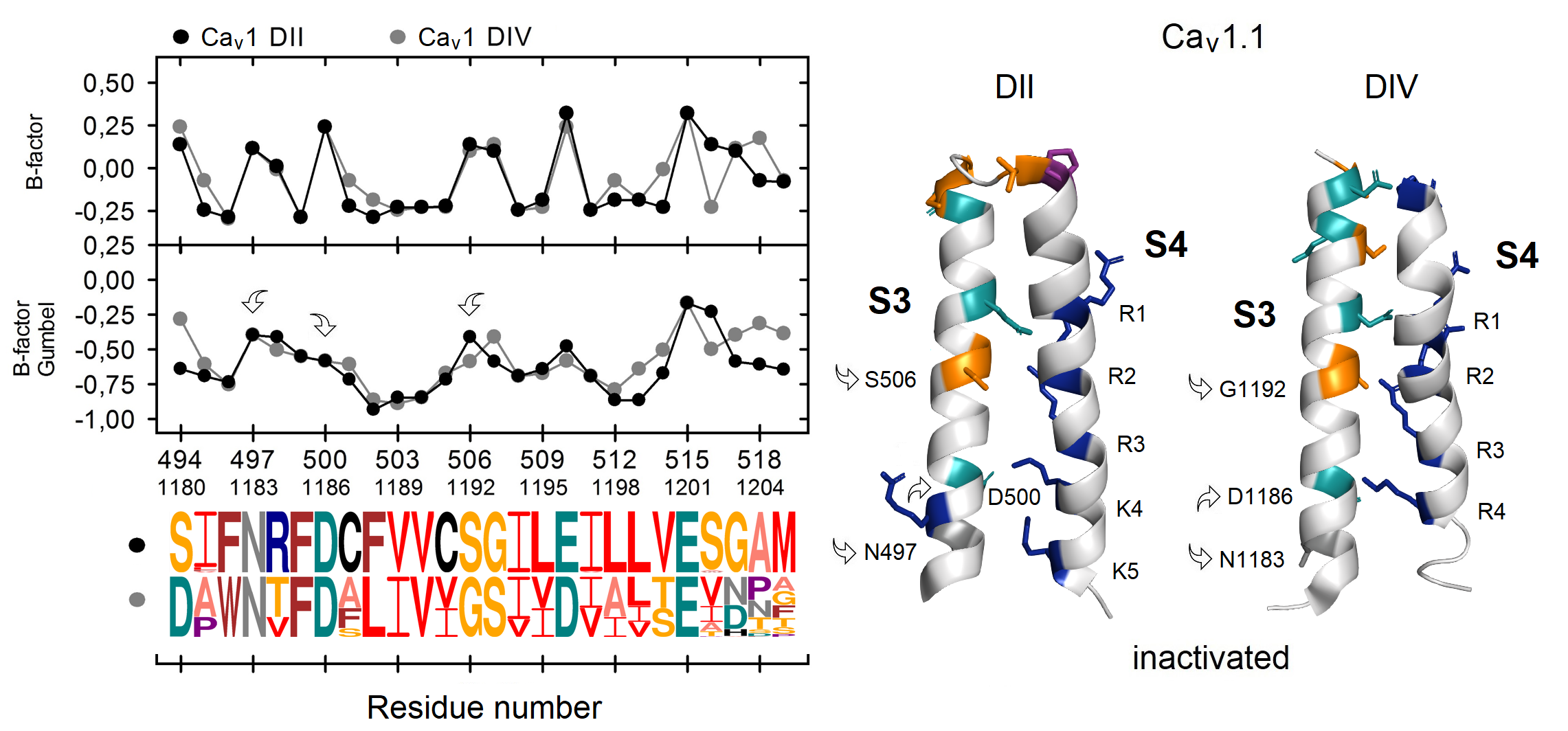

Supplement: Supplemental Material [file kchl-13-01-1674242-s001.zip › Supp-F6'_Cav1.png]

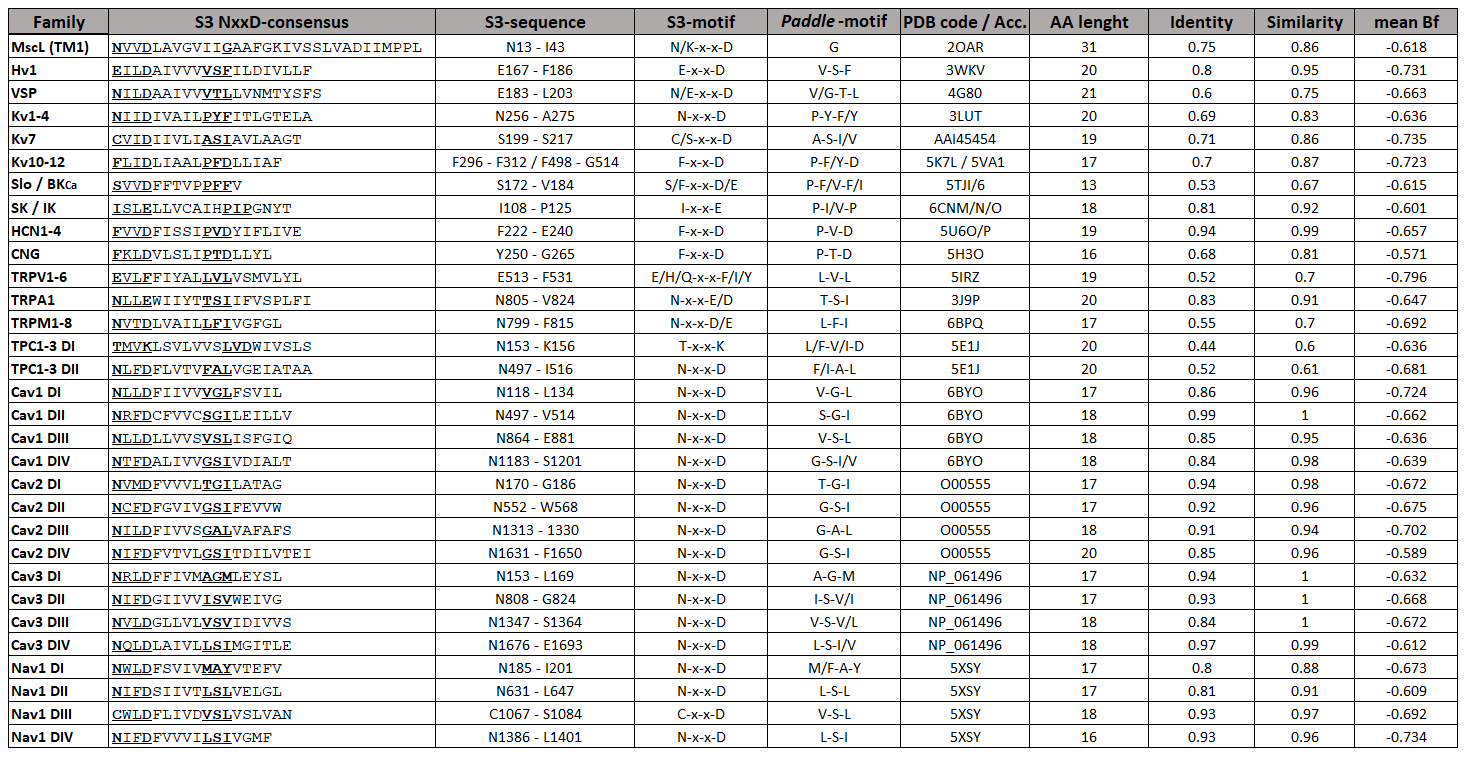

Supplement: Supplemental Material [file kchl-13-01-1674242-s001.zip › TABLE-S1.png]
